# Assessing the Impact of the Quality of Textual Data on Feature Representation and Machine Learning Models: Quantitative Study Using Large Language Models

**DOI:** 10.2196/73325

**Published:** 2025-12-30

**Authors:** Tabinda Sarwar, Antonio José Jimeno Yepes, Lawrence Cavedon

**Affiliations:** 1 Royal Melbourne Institute of Technology University Melbourne Australia

**Keywords:** aged care homes, clinical data, data quality, electronic health records, healthcare, large language models, machine learning, natural language processing, predictive modelling, error rate

## Abstract

**Background:**

Data collected in controlled settings typically results in high-quality datasets. However, in real-world applications, the quality of data collection is often compromised. It is well established that the quality of a dataset significantly impacts the performance of machine learning models. In this context, detailed information about individuals is often recorded in progress notes. Given the critical nature of health applications, it is essential to evaluate the impact of textual data quality, as any incorrect prediction can have serious, potentially life-threatening consequences.

**Objective:**

This study aims to quantify the quality of textual datasets and systematically evaluate the impact of varying levels of errors on feature representation and machine learning models. The primary goal is to determine whether feature representations and machine learning models are tolerant to errors and to assess whether investing additional time and computational resources to improve data quality is justified.

**Methods:**

We developed a rudimentary error rate metric to evaluate textual dataset quality at the token level. The Mixtral large language model (LLM) was used to quantify and correct errors in low-quality datasets. The study analyzed two health care datasets: the high-quality MIMIC-III public hospital dataset (for mortality prediction) and a lower-quality private dataset from Australian aged care homes (AACHs; for depression and fall risk prediction). Errors were systematically introduced into MIMIC-III at varying rates, while the AACH dataset quality was improved using the LLM. Feature representations and machine learning models were assessed using the area under the receiver operating curve.

**Results:**

For the sampled 35,774 and 6336 patients from the MIMIC and AACH datasets, respectively, we used Mixtral to introduce errors in MIMIC and correct errors in AACH. Mixtral correctly detected errors in 63% of progress notes, with 17% containing a single token misclassified due to medical terminology. LLMs demonstrated potential for improving progress note quality by addressing various errors. Under varying error rates (5%-20%, in 5% increments), feature representation performance was tolerant to lower error rates (<10%) but declined significantly at higher rates. This aligned with the AACH dataset’s 8% error rate, where no major performance drop was observed. Across both datasets, term frequency–inverted document frequency outperformed embedding features, and machine learning models varied in effectiveness, highlighting that optimal feature representation and model choice depend on the specific task.

**Conclusions:**

This study revealed that models performed relatively well on datasets with lower error rates (<10%), but their performance declined significantly as error rates increased (≥10%). Therefore, it is crucial to evaluate the quality of a dataset before using it for machine learning tasks. For datasets with higher error rates, implementing corrective measures is essential to ensure the reliability and effectiveness of machine learning models.

## Introduction

Electronic health records (EHRs) represent rich information capturing the heterogeneous aspects of an individual’s health, such as medical history, vital signs, prescriptions, laboratory tests, imaging reports, and treatment plans. Because of this, EHRs are now widely adopted in data-driven treatment approaches, where data mining and machine learning approaches have been applied for clinical applications [1-4]. A major part of the EHRs is in the form of free-text notes. This includes, but is not limited to, doctors’ notes, disease symptoms, nurses’ observations, findings from radiologists, and any adverse reactions to medication. This textual data, which encapsulates critical and complex information, has been extensively used in data mining and machine learning [5-12].

The growing importance of large-scale textual datasets has played a pivotal role in the development of transformer-based models, including large language models (LLMs) [13-17]. These models were initially developed using general-domain datasets (eg, Wikipedia, BooksCorpus, public GitHub repositories), which are not representative of biomedical text [17,18]. The biomedical corpus differs significantly from the general corpus due to its specialized vocabulary and concepts, limiting the application of general LLMs in the biomedical and clinical domains. Recognizing this limitation led to the development of domain-specific BERT (bidirectional encoder representations from transformers) models, such as BioBERT [19], ClinicalBERT [20], PubMedBERT [21], BioMed-RoBERTa [22], SciBERT [23], MedCPT [24], and GatorTron [25]. These models were trained on biomedical-domain corpora (PubMed abstracts and articles from PMC and Semantic Scholar) and open-source hospital datasets (MIMIC-III [26] and i2b2 [27,28]), except GatorTron, which used medical information from the private University of Florida Health Integrated Data Repository.

The data sources used for training biomedical or clinical versions of LLMs are considered high-quality datasets, with minimal grammatical, typological, and spelling errors. However, data collected from real-life applications often contain many such mistakes, including informal language use and nonstandardized abbreviations. It is well established that data quality significantly impacts the performance of machine learning and deep learning models [29-33]. Identifying errors in structured datasets is relatively straightforward due to their standardized and objective representation. Significant literature exists on assessing the quality of structured data [31,34-37], including aspects of EHRs [3,38-40]. These studies evaluated data quality across various dimensions, such as completeness, accuracy, integrity, validity, and consistency. In contrast, assessing the quality of textual data is more complex due to its subjective and unstructured nature. As textual data is increasingly used in daily routines, it is crucial to evaluate the impact of its quality on machine learning applications, especially in biomedical and clinical contexts. The quality of structured data has been extensively studied in the literature, along with its impact on machine learning algorithms [29,30]. However, the quality of unstructured textual data has not been thoroughly evaluated, particularly considering its challenging and subjective nature [29,33].

Several preliminary studies have explored methods for assessing the quality of textual data. Kiefer [41] suggested the following indicators for text data quality: (1) percentage of abbreviations, (2) percentage of spelling mistakes, (3) lexical diversity, (4) percentage of uppercased words, and (5) percentage of ungrammatical sentences. Larson et al [42] proposed a pipeline for dialogue systems to effectively detect two types of textual outliers: (1) errors, defined as sentences that have been mislabeled and can have a detrimental impact on the classification model, and (2) unique samples, defined as sentences that differ structurally from most others in the data but can enhance the model’s robustness. A few studies have not directly assessed the quality of textual data but have instead focused on understanding complex text classification tasks [43,44], improving models to generate diverse textual outputs and reduce the production of generic responses [45], and detecting noise in labels [46-49].

Given that the quality of textual datasets directly influences the training and performance of machine learning models, it is essential to assess how data quality impacts model outcomes. Despite the widespread use of text data in machine learning tasks, there is limited research focused specifically on the quality of textual datasets and its effect on model performance [50]. Swayamdipta et al [51] proposed “Data Maps” to identify textual samples that are easy to learn, hard to learn, and ambiguous for classification models, suggesting that hard-to-learn instances often correspond to data errors. Ribeiro et al [52] developed a checklist for testing natural language processing (NLP) models with respect to various aspects, including vocabulary, taxonomy, robustness to typos and irrelevant changes, named entity relationships, fairness, temporality, negation, coreference, semantic role labeling, and logic. Colavito et al [53] primarily assessed the quality of labels in classification tasks using pretrained language models (BERT, ALBERT, and RoBERTa).

A subset of the mentioned studies attempted to identify textual quality errors, but none conducted a systematic study to evaluate the value of investing extra effort in improving data quality and its association with the performance of machine learning models. Machine learning tasks involving medical textual datasets are more critical than those using other types of textual data (eg, social media, news articles) because they have a direct impact on human health and life. Therefore, it is essential to systematically evaluate the effects of erroneous low- and high-quality medical textual datasets on machine learning tasks in the health care domain.

To the best of our knowledge, this is the first study to systematically assess the quality of textual datasets and their impact on downstream machine learning models, addressing a critical and underexplored aspect of machine learning research. While the quality of structured datasets has been extensively studied for decades, the quality of unstructured textual data remains relatively neglected. Our work takes an important step toward filling this gap by highlighting how data quality affects model performance. We begin by defining and categorizing different types of errors that can occur in textual datasets. Artificial errors were then systematically introduced and controlled within the datasets to compare model performance across varying error rates. The study leverages both traditional (TF-IDF [term frequency–inverted document frequency]) and neural network (word2vec and BERT) embedding-based models to determine which models are more affected by poor-quality data. The generated embeddings were then used for the predictive tasks using state-of-the-art machine learning models (logistic regression, random forests, and XGBoost). We used publicly available (MIMIC-III [26]) and private (outsourced from Australian Aged Care Homes [AACHs]) health care datasets. The study focuses on biomedical data, emphasizing the importance of accuracy in this domain, as any error or bias in the data or models can have significant implications for health and patient outcomes. This is the first study to systematically evaluate the performance of NLP feature representation and machine learning models under varying levels of textual data quality.

## Methods

### Overview

**Figure 1:**
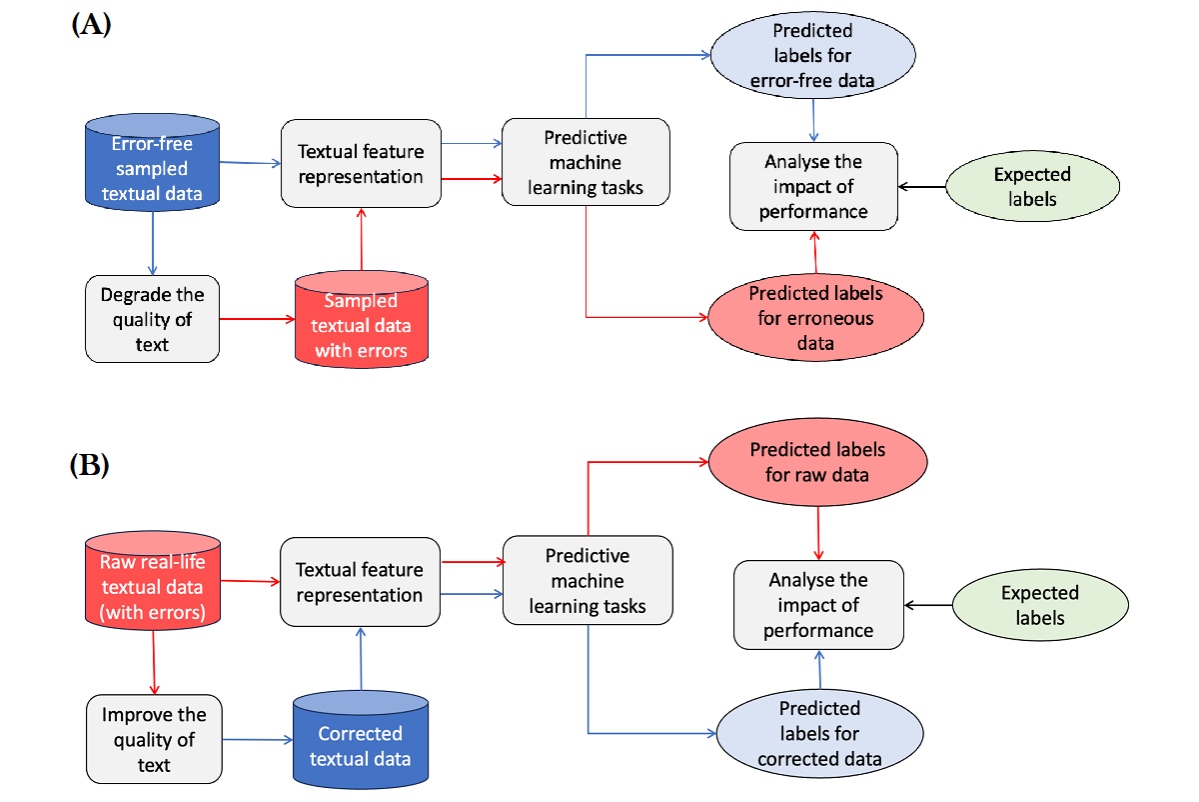
The study design for evaluating the impact of the quality of textual datasets on machine learning models. This involves two tasks: (A) systematically degrading the high-quality data (open-source MIMIC dataset) and (B) improving the low-quality data (acquired AACHs dataset). The high- and low-quality versions of the two datasets were then used for the feature representation and predictive models. The performance metrics of the predictive tasks for the two versions of datasets were analyzed to assess the impact of extent of errors on the feature representation and machine learning models. AACH: Australian aged care home.

In this study, we conducted a systematic analysis of the impact of textual dataset quality on NLP feature representation and machine learning models. The study design is illustrated in Figure 1. Briefly, we used medical datasets, both publicly available and private, representing high-quality (with no errors) and low-quality (with errors) datasets (section Datasets). We developed a pipeline to introduce errors into the clean data (section Data Processing). The LLM (specifically, Mixtral) was then used to process the low-quality data, correcting and removing errors. Both the processed and raw text datasets were subsequently used to generate feature vectors for classification tasks (section Selection and Evaluation of Feature Representation and Machine Learning Models). The outcomes for low- and high-quality datasets were systematically evaluated (section Results) to assess the impact of errors on the performance of machine learning models.

### Datasets

#### MIMIC Dataset

We used the MIMIC-III [26] publicly available medical database, which consists of deidentified health-related data of over 40,000 intensive care unit patients at the Beth Israel Deaconess Medical Center in Boston, Massachusetts, between 2001 and 2012. The database contains comprehensive information about the patients, including demographics, medications, diagnoses, medical procedures, caregiver notes, and admission and discharge summaries. As the study aimed to assess the impact of the textual dataset, we limited data selection to the PATIENTS, ADMISSIONS, and NOTEEVENTS tables of the MIMIC-III database.

#### Dataset From Aged Care Homes

AACHs regularly record resident data to comply with the quality standards set by the Australian Aged Care Quality and Safety Commission. This data captures multiple aspects of residents’ lives, including, but not limited to, demographics, medical history, medications, mobility, bowel movements, dietary requirements, and fall risk. A significant portion of the data consists of progress notes—free-text entries that document the daily progress or condition of residents. Nursing staff (both caregivers and registered nurses) routinely record the health status of older adult residents, often incorporating input from general practitioners.

The anonymized operational EHRs from AACHs, operated by a larger residential aged care provider, based in New South Wales, Australia, were acquired for this study. The dataset corresponded to 26 AACHs with a total capacity of 1160 beds from January 1, 2010, to January 1, 2021. Although the dataset comprised two broad categories—unstructured data (progress notes) and structured data (observation charts and assessment forms)—we used only the progress notes, incident reports, medical history, and demographic details for this study.

### Data Processing

To assess the impact of textual data quality on feature representation and machine learning models, datasets containing both erroneous and error-free text notes were needed to represent low- and high-quality data, respectively. To achieve this, we adopted two strategies described in the subsections Data Correction and Erroneous Data Generation. First, this involved defining and quantifying the textual errors in the dataset (subsections Types of Errors and Quantification of the Errors).

#### Types of Errors

The progress notes from MIMIC was deemed as an error-free dataset, as it has been extensively used in developing models and techniques in the medical and health care domain, and no textual mistakes have been reported in any study. On the other hand, we observed many errors in the progress notes in the AACHs dataset, including missing white space between words, grammatical errors, typological mistakes, spelling mistakes, and informal abbreviations (eg, “btw” instead of “between,” and “hwr” instead of “however”). Examples of these mistakes are reported in Table 1.

We required a robust mechanism to identify and quantify the extent of errors (mentioned in Table 1) in the dataset. The AACH dataset contained various types of errors within a single data instance, making it difficult to use existing tools like Autocorrect [54], PyAspeller [55], and Grammar-check [56] for error quantification, as these tools typically handle one type of error at a time. Their performance, particularly in identifying spelling mistakes, depends on a predefined dictionary. However, in the case of medical records, many specialized medical terms may not exist in the standard dictionaries used by such tools. Furthermore, many studies on improving text quality have often emphasized grammatical errors [57-59]. Recently, LLMs have been applied to correct grammatical mistakes in textual datasets, making them the most advanced tools available for improving text quality [57,59].

Many LLMs can be found in the literature [14], but considering the trade-offs between computational complexity, availability (open source), and performance [60,61], we selected Mixtral 8x7B for this study [62]. We conducted a preliminary study to explore the use of different LLMs for correcting textual errors (subsection Data Correction). However, a comprehensive evaluation of various LLMs for dataset correction is beyond the scope of this study and is left for future work.

**Table 1:** Sample of errors found in the textual dataset acquired from aged care homes. Text linked with any identifiable information was removed from these samples. The italicized text indicates the errors in the textual samples.

Types of error	Textual samples
Spelling mistakes	“Staff spoke to XYZ regarding her eye lashes. XYZ to arrange a time with optomristrist” “Resident has had very little sleep overnite” “Resident was i/c of urine when staff went to check on her after her bed sencer went off”
Typological mistakes	“Resident found sitting in chair on 0400hrs round check. Was tioleted and directed back to bed. Given water on both occassions” “Assisted x 2 staff with ADLs and personal hygiene. Transfered with stand-up lifer”
White space mistakes	“Resident sitting in the lounge area with fellow residents,while sitting” “Resident laying on bed feeling unwell.Resident wanted to go to the toilet.Staff assisted Resident” “Antibiotics conti nue for infected toe - dressing remains intact this shift.”
Grammatical mistakes	“Resident was found incontinent of faeces while sitted in lounge chair.” “she is look so confused.” “XYZ has participate with mainstream activities during the day.”
Informal abbreviations	“XYZ given. resident being reviewed by GP tmw for her pain and a suitable pain regime.” “Resident came down for bfast and ate all of her maeal.”
Miscellaneous	“Post lunch staff intervention/ s required as resident became verbally distruptive w/ another resident.” “XYZ is also walked to crafts,and atttend as many group activities as she can,to,decreased isolation.”

#### Quantification of the Errors

To quantify the extent of errors and assess their impact on prediction tasks, we tested Mixtral across different categories of errors (Table 1) to assess its performance. We conducted similar tests using the proprietary GPT-4 model, which is considered one of the most advanced LLMs compared with currently available open-source models. We found that the LLMs struggled to differentiate between various types of errors, such as distinguishing spelling mistakes from grammatical errors. For example, when asked to correct only grammatical mistakes, the LLM would also correct any spelling mistakes. To address the limitation of distinguishing between different types of errors, we developed a generalized metric for error quantification. This approach involved splitting each textual data instance into tokens based on white space and evaluating each token for errors. Hence, the error rate was defined as:



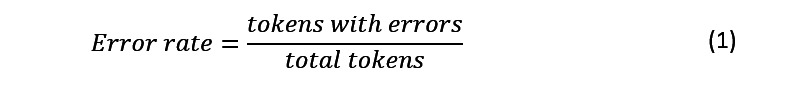



This metric disregarded punctuation and grammatical errors, focusing instead on missing white space between words and spelling mistakes (Table 1), which were frequently observed in the AACH dataset. The error rate was computed at the instance level, and the average was computed for the complete dataset.

This quantification was important, as the study aimed to assess the impact of varying error rates on feature representation and machine learning models. The mentioned error rate was used to synthetically add errors to the MIMIC dataset (subsection Erroneous Data Generation) and to quantify the extent of errors found in the AACH dataset.

To quantify the error rate, the AACH data instances were tokenized on white space, and a query was designed for Mixtral to identify the tokens with errors. The prompt and Mixtral settings used for the experiments are provided in Table S1 in Multimedia Appendix 1, along with an example in Table S2 in Multimedia Appendix 1. The response from Mixtral was used to quantify the error rate of the dataset, serving as a baseline for errors commonly found in low-quality, real-world datasets.

It is well known that the performance of LLMs is not perfect; that is, the outcomes generated by these models may contain errors. In this case, Mixtral might detect an error that does not exist or miss an actual error in the data. To evaluate the accuracy of LLM error quantification, we randomly sampled 150 data instances from the AACH dataset. These 150 instances were manually corrected to create a high-quality, error-free dataset, referred to as the “ground truth” for this study. The error rate was computed by Mixtral for this ground truth dataset using the aforementioned methodology. Analyzing these results helped assess Mixtral’s performance in detecting errors in the dataset.

#### Data Correction

As previously mentioned in the subsection Types of Errors, traditional tools and methods are not suitable for correcting text in cases involving out-of-dictionary words and missing white space between words. Therefore, we used Mixtral to correct these errors. The correction query used in the study is provided in Supplementary Tables S1 and S3 (Multimedia Appendix 1). This query specifically addresses spelling mistakes to maintain consistency with the error quantification outlined in subsection Types of Errors. Mixtral also corrected grammatical mistakes in many instances, a trait observed with other LLMs as well.

The ground-truth dataset was corrected using Mixtral. This correction was then qualitatively compared with the manually corrected data instances to assess the performance of Mixtral in correcting textual errors within the dataset, with details reported in section Results.

Although Mixtral was primarily used for error correction across the complete dataset, we also sampled a subset of the MIMIC dataset and applied corrections using Llama 3.3, Gemma 3-4B-IT, and DeepSeek-R1-Distill-Qwen-1.5B. The various corrected versions of the dataset generated by these models were then used for prediction tasks. This was done to compare the impact of different LLMs on error correction quality and, subsequently, on the performance of machine learning models. We focused on open-source models for this study; however, a comprehensive comparison of LLMs for linguistic error correction is beyond the scope of this study.

#### Erroneous Data Generation

In order to assess the impact of textual dataset quality on feature representation and machine learning models, we required a controlled generation of textual data with varying levels of errors. To achieve this, we introduced artificial errors into the MIMIC dataset corresponding to varying error rates. The study focused on two types of errors: misspellings and missing white space between words. The proportion of these two error types was randomly determined based on the overall error percentage. For example, in a dataset with 10% errors, this percentage could be randomly split, such as 4% for misspelled words and 6% for missing spaces. This was calculated using equation (1), where the number of erroneous tokens or words was computed based on the total number of tokens in a given textual data instance for a given error rate.

To generate misspelled versions of words, we used Mixtral to create incorrect counterparts. For introducing missing white space errors, we placed more emphasis on removing spaces between words separated by punctuation marks, as this pattern was observed in real-life datasets (Results section). To achieve this, the probability of sampling words containing punctuation marks was 50% higher than that of tokens without punctuation when randomly selecting tokens for removing white space.

It should be noted that the errors were introduced at the token level using Mixtral. Therefore, even if a different LLM model was used for this purpose, the overall error rate would remain the same, although the nature of the spelling mistakes might differ.

### Adverse Events and Data Sampling

We used different adverse events predictions for the two selected datasets to assess performance across a broad range of tasks, rather than focusing on a single specialized task. The sampled textual notes for each individual were concatenated to form a single input for the prediction model.

#### MIMIC Dataset

The mortality prediction task was selected for the MIMIC dataset, using progress notes recorded within the first 24 hours of intensive care unit admission to predict patient outcomes. The task is binary, with the outcomes classified as “alive” (represented by a value of 0) and “deceased” (represented by a value of 1). Detailed information about the data sampling process, including inclusion criteria, preprocessing steps, and any filtering applied, can be found elsewhere [63].

#### AACH Dataset

For the AACH dataset, we focused on predicting the risks of depression and falls among older adults. The main objective was to evaluate performance on high- and low-quality textual datasets, rather than focusing on mortality prediction in hospital and aged care settings.

Residents with depression or having a fall incident were considered as case 1 for binary classification, and the absence of the adverse event was considered as 0. Due to intellectual property (IP) concerns related to this solution, which is currently being deployed for the AACHs, we are unable to include the details of the data sampling here. However, we ensured that the sampled data was used to predict adverse events, both in the presence and absence of textual errors. The results of this analysis are reported in the Results section.

### Selection and Evaluation of Feature Representation and Machine Learning Models

#### Feature Representation

In the following sections, we describe two methods to derive features: traditional TF-IDF and deep learning–derived embedding features.

#### TF-IDF Features

Traditionally, text has been split into tokens, and the tokens have been used as features in machine learning algorithms. We used the following regular expression to split the text: (?u)\b[a-zA-Z]+\b. After converting the tokens to lowercase, the term frequency per document and the inverted document frequency were calculated for each token in a document (implemented using the scikit-learn Python library). We used unigram-level TF-IDF features to assess the results at the token level.

After computing TF-IDF features and removing the stop words, we further performed the following selection of tokens: (1) top tokens—TF-IDF (top): selected the top 5000 most frequent tokens; and (2) tokens with a minimum frequency threshold—TF-IDF (min): selected tokens that had a frequency greater than 5. This threshold could be changed, but we chose a frequency of 5 to remove the comparatively most unique words found in the corpus. Testing different threshold values is beyond the scope of this study.

### Embedding Features

Skip-gram word2vec embeddings: For tokenization, we used the same strategy used for generating TF-IDF features, which involved applying the specified regular expression followed by converting tokens to lowercase. The generated tokens were matched against the dictionary of the word2vec-google-news-300 model [64], which was pretrained using 3 million words and phrases from Google News and has a vector dimension of 300. If a token was not matched in the dictionary of the trained model, it was skipped and not considered as a feature. Feature vectors generated for each token in a textual data instance by the word2vec model were concatenated and averaged to calculate an embedding feature for the data instance.

BERT embeddings: BERT [65] has two advantages compared with word2vec. First, the tokenization relies on word-piece tokenizer that splits words into subwords, which can increase the matching of tokens to embeddings, unlike word2vec, which ignores out-of-vocabulary tokens. Second, word2vec generates static embedding; that is, each word has a single fixed vector, whereas BERT generates an embedding vector that considers the context of a word.

There are various variants of BERT models such as Clinical BERT [66], BioBERT [19], ClinicalBERT [20], PubMedBERT [21], BioMed-RoBERTa [22], and LongFormer [67], but several issues arose when applying these models directly to this study. The length of tokens in the sampled progress notes in the AACH setting exceeded the token limits set by BERT (512 tokens) and LongFormer (4096 tokens). Truncating the progress notes to accommodate these limits resulted in poor performance of classification tasks. To address this limitation, an embedding vector (768 dimensions) for each token was computed using the BERT model. The embedding vectors for tokens in a progress note were concatenated and averaged to calculate the final feature vector for the classification task. We used the BERT model pretrained on clinical data (ClinicalBERT) [66] and BioClinical ModernBERT [68] for the study.

### Machine Learning Models and Evaluation

Adverse event prediction was modeled as a classification problem, in which 0 indicates no risk of an adverse event and 1 indicates otherwise. We selected logistic regression, Support Vector Machine Classifier, random forests, XGBoost, and Multilayer Perceptron (MLP) Classifier for the study. Numerous machine and deep learning models are available, but an elaborate evaluation of these models is beyond the scope of the study. The goal of the analysis was to assess whether the quality of the dataset impacts the performance of machine learning models. Hence, identifying the model that is least or most affected by data quality was not the study’s primary goal. We used two-thirds of the data for training and one-third for evaluation.

The performance of the models was primarily evaluated using the area under the receiver operating curve (ROC-AUC). It is important to note that our primary evaluation metric is ROC-AUC, a widely used measure for assessing the overall performance of machine learning models, particularly in binary classification tasks. Although precision, recall, and *F*_1_-score were also computed, they were not used as the primary basis for comparison. Our objective was not solely to optimize classification accuracy for a specific class, but rather to evaluate how data quality impacts the overall predictive performance of machine learning models. Precision, recall, and *F*_1_-score are suited for studies where the aim to analyze false positives or false negatives is high. In contrast, ROC-AUC provides a threshold-independent evaluation and reflects the model’s ability to distinguish between classes across all thresholds, providing a trade-off between sensitivity and specificity. This makes ROC-AUC an appropriate metric for this study to assess impact of data quality on model performance.

Hyperparameter tuning was performed for the selected models, and the best results based on ROC-AUC scores are reported here (parameters are provided in Tables S9-S18 in Multimedia Appendix 1). Results for different hyperparameter settings and secondary evaluation metrics are also provided in Tables S9-S18 in Multimedia Appendix 1.

We emphasize that we did not train a transformer model for the downstream task. Current transformer-based models are unable to handle long textual data, making it infeasible to process lengthy clinical notes in their entirety without truncation. Therefore, we focused on using embeddings from transformer-based models (subsection Embedding Features).

### Ethical Considerations

The Human Research Ethics Committee of the Royal Melbourne Institute of Technology University (Project 23257), Australia, provided ethics approval for this study. Informed consent was obtained from the administration and management of the aged care homes, who had obtained informed consent from the residents or their legal guardians. We did not receive approval to share the details of sampled data for AACHs. The dataset was deidentified prior to its use in analytical and predictive tasks.

## Results

### Descriptive Analysis

#### MIMIC-III and AACH Datasets

MIMIC-III contains anonymized data for 46,520 patients, encompassing 58,976 hospital admissions. Details of the sampling strategy (inclusion and exclusion criteria) can be found in a previous study [63]. This study focused only on predicting mortality using the first 24-hour window after admission, whereas the previous study [63] assessed multiple prediction windows. The descriptive analysis of the sampled data used in this study is reported in Table 2. As mentioned previously, we did not receive the approval to share the details of sampled data for AACHs.

**Table 2:** Characteristics of the selected residents for predicting mortality for the MIMIC dataset.

Characteristics			MIMIC: mortality risk
Total sampled patients, n			35,774
Age (years), mean (SD)			62.5 (16.5)
**Gender, n (%)**			
	Male	26,993 (42.7)	
	Female	20,148 (57.3)	
Death events (total), n (%)			4221 (11.8)
**Death cases by gender, n (%)**			
	Male	2344 (6.6)	
	Female	1877 (5.2)	

#### Ground Truth and Processed Datasets

**Figure 2:**
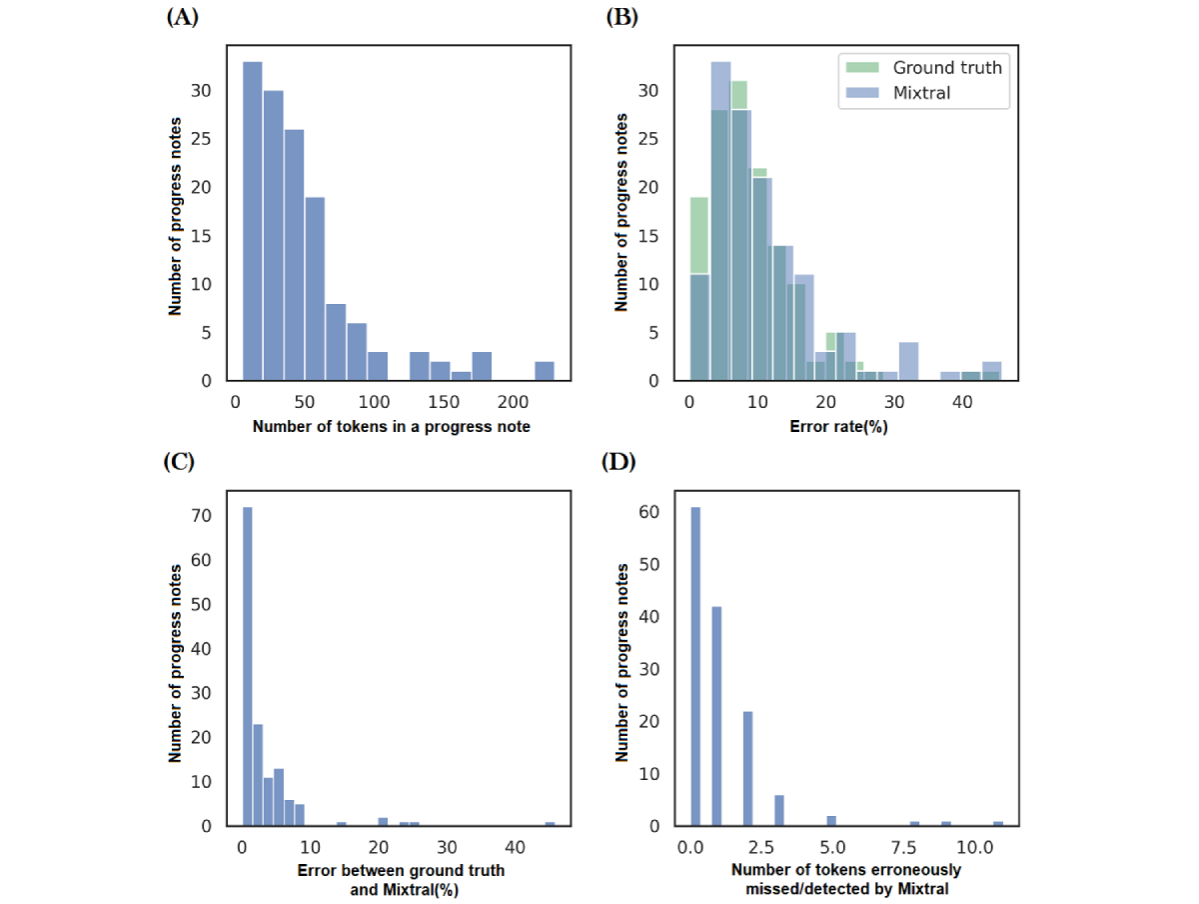
Mixtral’s performance on 136 ground truth AACH progress notes for quantifying the textual errors. (A) Number of tokens found in the ground truth progress notes. (B) Comparison of the error rate (%) found by Mixtral with the ground truth error rate. (C) The absolute error between the Mixtral-computed and ground truth error rate (%). (D) Number of tokens that were erroneously detected or missed by Mixtral. AACH: Australian aged care home.

As mentioned in subsection Quantification of the Errors, we first assessed Mixtral’s performance in quantifying errors in the textual dataset. For this purpose, we randomly sampled 150 progress notes from the AACH dataset and excluded progress notes with fewer than 5 tokens to ensure that very short progress notes are not included in the analysis. This exclusion was based on observations from the AACH dataset, which indicated that short progress notes did not contain any errors. The remaining 136 progress notes were manually assessed to quantify the extent of errors; following the procedure described in subsection Quantification of the Errors, each progress note was divided into tokens, and erroneous tokens were manually quantified. This served as the ground truth used to assess the Mixtral’ performance for error quantification, with results reported in Figure 2.

A performance deviation of 2.95% (SD 5.67%; actual number of mismatched tokens, 1.04, SD 1.61) was observed for Mixtral from the ground truth. Qualitative analysis of the results revealed that Mixtral erroneously identified medical terminologies (eg, ATOR, ADL, Perianal, situ, mane, obs, RN, etc) as misspelled tokens. Moreover, a few cases of hallucinations were also observed; for example, Mixtral hallucinated the misspelling “*angly*” from the following progress note, “Resident became angry during an activity.” Another instance involved Mixtral hallucinating the misspelling “refuced” from the progress note, “She refused to get out of bed and the staff tried many times to coax her out of bed to no avail.” This analysis demonstrated that Mixtral overestimated errors in some cases. Overall, Mixtral correctly identified misspelled tokens for 45% of the progress notes, and 17% of the progress notes contained only one erroneously identified or missed token.

The error rate for the AACH dataset, considered poor-quality data (subsection Types of Errors), was found to be 7.5% (SD 8.2%; Figure S1 in Multimedia Appendix 1). Considering this as a baseline of errors found in the real-world dataset, synthetic errors from 5% to 20% in increments of 5% were added to each progress note of the MIMIC dataset. This helped in the controlled simulation of poor-quality datasets, so we could systematically compare any change in the performance of the feature extraction and machine learning models with respect to the high-quality dataset (original MIMIC dataset in this case).

Furthermore, to evaluate Mixtral’s performance in correcting errors, we used the ground-truth dataset containing the real-world errors found in the AACH dataset and processed it through Mixtral. The corrected dataset was then used to quantify the proportion of errors that remained uncorrected (subsection Quantification of the Errors). We observed that the error rate was reduced from 7.5% to 3.4% in the corrected version, indicating that approximately 54% of the original errors were successfully corrected by Mixtral. This demonstrates that a substantial number of errors are corrected by Mixtral, thereby improving the overall quality of the dataset. It also highlights that selecting a more powerful LLM can further enhance the quality of textual data. We have used other open-source models for textual dataset correction and its impact on the predictive tasks (section Comparative Evaluation of LLMs for Data Correction).

It is important to note that Mixtral may also correct some grammatical errors (Table S3 in Multimedia Appendix 1). However, due to the inherent complexity of grammatical error correction, this aspect is not captured in the proposed rudimentary error rate. Further discussion on this limitation is provided in the Discussion section.

### Feature Representation and Machine Learning Models

In this section, we present results comparing the performance of the machine learning classifiers trained on several feature representations of the progress notes with different levels of errors.

#### Mortality Prediction

**Figure 3:**
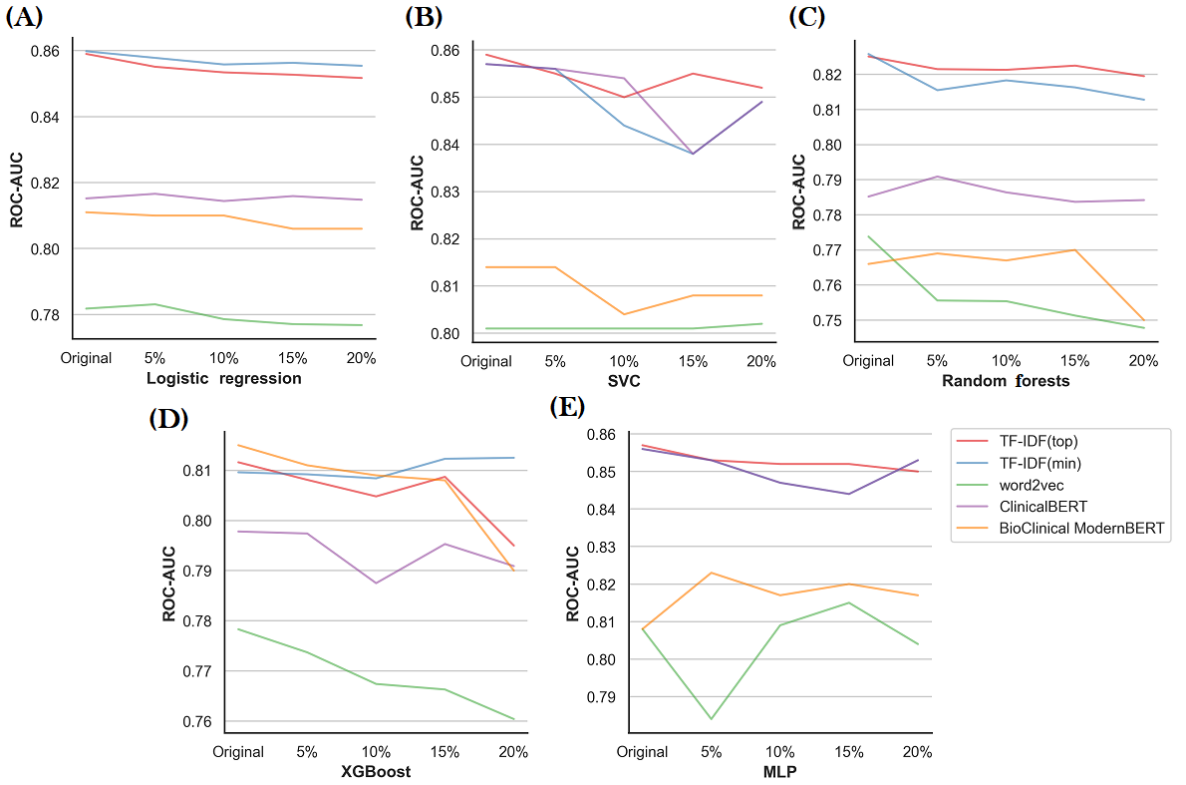
The performance of machine learning models for varying quality of the MIMIC dataset using different feature representation techniques. MLP: Multilayer Perceptron; ROC-AUC: area under the receiver operating curve; SVC: Support Vector Machine Classifier; TF-IDF (min): tokens with a minimum frequency of 5; TF-IDF (top): top 5000 most frequent tokens.

The results for mortality prediction using different feature representations and machine learning models are presented in Figure 3. We found that logistic regression had comparatively higher ROC-AUC than other machine learning algorithms for the majority of the feature representation models. In terms of the feature representation models, TF-IDF models outperformed neural network–based embedding models (word2vec, ClinicalBERT, and BioClinical ModernBERT).

Figure 3 clearly illustrates that, as hypothesized, the performance of the trained models declined as the level of errors in the dataset increased. Although the reduction in ROC-AUC is not substantial, even small variations can have a significant impact in critical domains such as health care and biomedicine.

#### Depression and Fall Prediction

For the AACH dataset, we predicted fall and depression for both the original (low-quality progress notes with errors) and corrected (high-quality) sampled datasets. A substantial difference between the two datasets was not observed, with the difference in ROC-AUC values ranging from 0.002 to 0.004. This finding is consistent with the results reported in the subsection Mortality Prediction. A substantial difference in decline of performance for MIMIC data was observed at error rates ≥10%, whereas the average error rate of AACH dataset is 7.5% (subsection Ground Truth and Processed Datasets).

In terms of machine learning models, logistic regression had the lowest performance for the classification tasks. Similar to mortality prediction (subsection Mortality Prediction), the traditional TF-IDF feature representation model outperformed the embedding models. We are unable to share the precise details of the results due to IP constraints enforced by the participating AACHs.

### Comparative Evaluation of LLMs for Data Correction

We primarily used Mixtral for error correction and evaluated its impact on feature representation and the performance of machine learning models. As it is well established that LLM performance may vary, often including biases and errors, with some models outperforming others in specific tasks. This applies to Mixtral as well, where using a different model might yield different outcomes in correcting textual errors, in turn having varying performance on machine learning tasks. To assess the performance of various LLMs in error correction, we used 3 open-source models: Llama 3.3, Gemma 3-4B-IT, and DeepSeek-R1-Distill-Qwen-1.5B.

For this evaluation, we sampled 6750 patients (approximately 5% of the original MIMIC dataset), corresponding to 40,000 progress notes. The sampling was done while preserving the distribution of prediction labels to ensure that the subset remained representative of the full dataset. This sampling approach was necessary because the full dataset contains over a million progress notes, with each progress note averaging around 2000 tokens, making it computationally expensive to process the complete dataset with multiple LLMs. Therefore, while Mixtral was used to correct the entire dataset for the primary analysis, the smaller sample was used to compare the performance of different LLMs.

**Figure 4:**
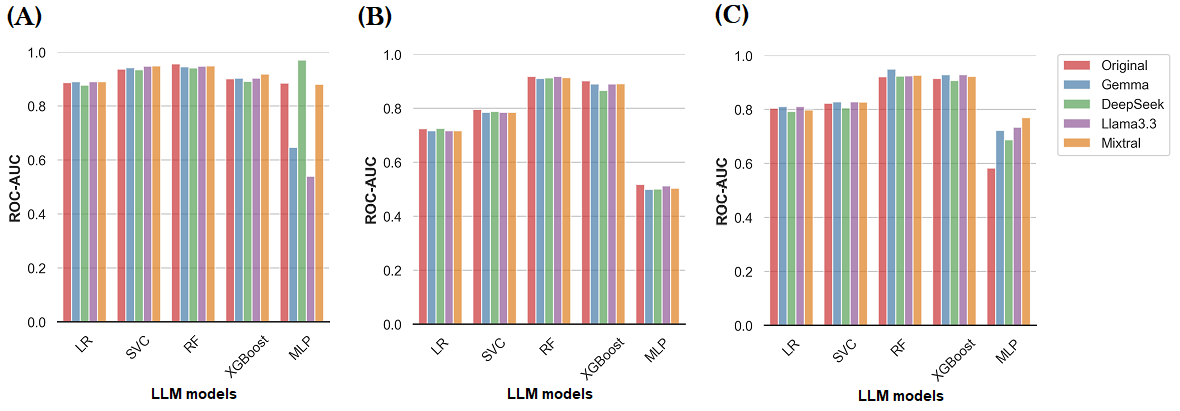
The performance of machine learning models for the MIMIC dataset with 10% error-rate corrected using different large language models (LLMs) for three representative feature representation techniques, namely (A) term frequency–inverse document frequency (TF-IDF [min]; tokens with a minimum frequency of 5), (B) word2vec, and (C) BioClinical ModernBERT. LR: logistic regression; MLP: Multilayer Perceptron; RF: Random Forest; ROC-AUC: area under the receiver operating curve; SVC: Support Vector Machine Classifier.

To ensure a fair evaluation of LLM-based correction, we used the generated erroneous version of the MIMIC dataset containing 10% token-level errors. This was chosen to resemble real-world error distributions (subsection Ground Truth and Processed Datasets). This setup allows a fair comparison between machine learning performance on the LLM-corrected datasets and the original, error-free MIMIC dataset. The results are reported in Figure 4. We observed comparable performance across different LLMs, which may be attributed to the fact that the downstream machine learning models are generally tolerant to error rates of up to 10% (section Feature Representation and Machine Learning Models). As a result, any LLM that improves the quality of the textual dataset compared to the original error-prone version may yield similar performance. This could be explain why a major difference between the performance of the models under varying LLMs was not observed. It is also worth noting that the slightly lower results observed with some MLP classifiers (Figure 4) could potentially be improved through further hyperparameter tuning.

## Discussion

### Overview

The performance of the machine learning models depends on the quality of the dataset used. Although the quality of the structured data has been extensively studied (section Introduction), this study aimed to quantify the impact of the quality of unstructured textual data on machine learning tasks. This is crucial, as it demonstrates whether investing additional time and computational resources to enhance data quality is truly worthwhile.

Identifying errors in structured data is comparatively easier than in unstructured textual datasets due to its objective nature. However, the subjective and contextual characteristics of textual data make defining and identifying different types of errors (Table 1) a challenging task. In the section Introduction, we discussed recent studies that have attempted to identify various quality issues in text, but this remains an open research question. Given its complexity, we focused on identifying errors at the token level (specifically spelling mistakes and missing white space) rather than at the sentence level (eg, grammatical mistakes). This approach allowed us to quantify the error rate as the ratio of the number of tokens with errors to the total number of tokens. This metric was crucial for evaluating the impact of data quality on the performance of feature representations and machine learning models.

Our rudimentary error quantification metric covers spelling mistakes and missing white space, but other types of errors (“informal abbreviations” and “typological mistakes,” along with those listed under the “miscellaneous” category) can also be broadly covered under the same criteria at the token level. Quantifying grammatical issues in text is challenging due to the complex, context-dependent, and often subjective nature of language. Moreover, sentences can be ambiguous or only marginally incorrect depending on usage, dialect, or formality, making it difficult to identify the extent of grammatical errors. For example, in the case of typological mistakes, it is easy to quantify errors at the token level, which is not the case for grammatical errors. Grammatical errors can occur at the sentence level, where medical progress notes may be a combination of multiple sentences. Even minor mistakes, such as a determiner-noun mismatch, raise the question of whether they should be considered errors. Additionally, grammatical errors often overlap (eg, verb tense and subject-verb agreement), making it difficult to isolate and count them precisely. Hence, treating such errors as binary at the sentence level is challenging, as even a small mistake can lead to saturation of the metric. Moreover, human judgments about grammar can vary, and even LLMs are prone to errors [57]. Considering these challenges, we have limited the error quantification metric to two types of errors.

This study was designed to systematically assess the impact of textual dataset quality on feature representation and machine learning under two scenarios: (1) using a high-quality dataset and introducing varying levels of errors, and (2) using a low-quality dataset and correcting errors to improve its quality. For this purpose, we used the publicly available MIMIC-III high-quality dataset and an outsourced, real-world lower-quality dataset from AACHs. To introduce and correct errors, we used Mixtral, as LLMs are increasingly used to identify various issues in textual datasets (section Introduction). While LLMs have demonstrated superior performance across many tasks [14,60,61], they are not perfect.

To evaluate Mixtral’s performance in identifying errors, we created a manual ground-truth dataset of 136 progress notes from the AACH dataset. This effort was necessary to ensure Mixtral’s reliability in quantifying erroneous tokens, given that traditional dictionary-based tools often fail to detect errors such as missing white space between tokens, which was frequently observed in the AACH dataset (Table 1). We found that Mixtral correctly detected errors in 63% of the progress notes, with 17% containing a single token mistakenly identified as an error. Qualitative analysis revealed that Mixtral misclassified medical terms as errors, leading to an overestimation of the error rate (subsection Ground Truth and Processed Datasets). After evaluating Mixtral’s performance, we used it to systematically introduce errors into the MIMIC-III dataset and to quantify the error rate of the AACH dataset (subsection Ground Truth and Processed Datasets).

We also used Mixtral to correct errors in the AACH dataset to improve its quality. Unlike using the error rate at the token level, we provided the unprocessed progress notes as input for error correction (Table S3 in Multimedia Appendix 1). When assessing Mixtral’s performance in error correction, we found that incorporating the context of the sentence led to better correction of errors compared to token-level corrections. Examples demonstrating Mixtral’s poor performance on token-level error correction are provided in Table S4 in Multimedia Appendix 1. Sentence-level corrections not only resolved token-level errors but also addressed grammatical errors. While we acknowledge that grammatical mistakes were not a focus of this study, it is worth noting that no major grammatical issues have been reported in the MIMIC dataset. Therefore, the corrected AACH data represented a high-quality dataset.

Tables S5 and S6 in Multimedia Appendix 1 represent the impact of errors on the tokens extracted from the progress notes. Interestingly, as the error rate increased, the number of unique tokens also increased, while the average length of the notes decreased. This could be the result of the removal of white space and the addition of misspelled tokens to create a low-quality dataset.

We used several methods to generate the features used for the machine learning models. These features were derived from the text of the records in both datasets. Table S7 in Multimedia Appendix 1 shows the number of features in the MIMIC dataset depending on the feature representation and the error rate. It can be observed that the number of features increases as soon as the percentage of errors increases for the TF-IDF features. This is due to the unique erroneous tokens that increased with the error rate. If no filtering is applied on the TF-IDF features, then there are 10 times more unique features compared to the original set, while it is 5 times in the case of the filtering of features by frequency.

Among the different machine learning models, logistic regression had comparatively the best performance for the mortality prediction task using the TF-IDF–derived features. It is evident from Figure 3 that the performance of the prediction models decreased with the increase in the error rate. Although the performance in terms of ROC-AUC is tolerant to small error rates (<10%), it starts to be significant with a high error rate (≥10%). From Figure 3, it can be observed that neural network–based embedding techniques with XGBoost are more sensitive to noise, as their performance deteriorated for smaller error rates (<10%). The MLP did not exhibit a consistent trend of performance degradation with increasing error rates. This can be attributed to the inherent robustness of neural networks, which are known to be more tolerant to noise in the input data due to their distributed representations and nonlinear learning capabilities.

Table S8 in Multimedia Appendix 1 shows the statistics for the different partitions for the tasks for the AACH dataset. The number of unique features increases with the number of days, as a greater number of progress notes are generated over longer periods. It should be noted that, despite the progress notes being longer, the proportion of additional unique words in the AACH progress notes was smaller compared to the MIMIC dataset. This might be due to the limited dictionary of AACH, that is, the smaller number of unique words irrespective of the length of the progress notes. This implies that the MIMIC dataset had a comparatively diverse dictionary. The AACH dataset includes daily notes from aged care residents and reports on activities and findings of the residents, which might show more repetitions of similar progress notes compared to the MIMIC notes. This might explain why we did not see a large difference with the corrected notes in terms of unique terms. It should be noted that a substantial difference between the original and corrected AACH dataset was not observed (the results could not be shared due to IP concerns). This is consistent with the finding of MIMIC dataset (Figure 3), where a major impact for many models was not observed for an error rate <10%.

For the MIMIC dataset, linear regression shows higher prediction performance with TF-IDF features, which might indicate that individual words have higher predictive power for the mortality task. This seems consistent with the result of experiments with higher error rates, as errors were introduced on the token level (Figure 3). We followed Mahbub et al [63] for predicting mortality, who also reported lower performance of deep learning–derived features. Among embedding-based techniques, we found that BERT models outperformed word2vec (Figure 3). As mentioned in subsection Feature Representation, word2vec ignores out-of-vocabulary words, which can lead to the loss of valuable information contained in these errors. BERT models, on the other hand, address this limitation by subdividing words into subwords and using these subwords to generate embeddings. Consequently, BERT retains comparatively more information. Furthermore, the BERT model considers the context of each word within its surroundings. These properties likely contributed to BERT’s superior performance compared to word2vec. We used pretrained versions of word2vec, ClinicalBERT, and BioClinical ModernBERT, as retraining these models is computationally expensive. Additionally, retraining on erroneous datasets could lead the models to recognize erroneous tokens as valid, thereby nullifying the need to assess the true impact of errors on machine learning performance.

Generally, embedding techniques involving longer text, such as mortality prediction for MIMIC clinical notes, present significant architectural challenges. These clinical notes often exceed the typical 512-1024 token limits of transformer models, requiring truncation strategies that inevitably lead to information loss. Splitting the text into chunks and averaging their embeddings further contributes to task-specific discriminative information and contextual loss, which undermines the core strength of transformer-based models [65,69]. In contrast, TF-IDF inherently handles variable-length documents without such limitations, offering a more flexible approach in this regard. Furthermore, clinical prediction tasks often rely on specific medical terminology, drug names, and diagnostic codes that serve as strong predictive signals. TF-IDF captures these explicit keyword matches directly, whereas embeddings may dilute these signals by grouping semantically similar but contextually distinct terms. Additionally, it is possible that some of these critical terms are not part of the pretrained model’s vocabulary, further limiting the effectiveness of embedding-based approaches in such cases

In classification tasks where the presence or absence of specific words is critical, as in the case of mortality prediction where particular symptoms, medications, or vital signs are highly predictive, traditional sparse features often produce more linearly separable feature spaces than complex embeddings [70]. We observed this in this study as well, where linear models outperformed more complex embedding models. This finding aligns with recent work showing that traditional methods remain competitive in clinical tasks where specific lexical features carry more predictive power than broader semantic relationships [63,69,70].

For the AACH dataset, the predictive performance of XGBoost was higher compared to linear regression using TF-IDF features (the detailed results could not be shared due to IP concerns). It is well-established that no machine or deep learning model performs optimally across all tasks. This is also evident from our experiments, where the performance varied across different feature representations and machine learning models for the two tasks.

Overall, we found that the feature representation and machine learning models are tolerant of small errors (<10%). The real-world AACH dataset contains errors (approximately 8% in this case), but the performance of the prediction tasks can be optimized by careful selection of the feature representation and machine learning models. For the case, when the error rate is large (≥10%), corrective measures are necessary to ensure good performance of the predictive models.

### Limitations

There are a few limitations to this study that should be acknowledged. First, the error quantification metric we used was rudimentary and did not account for the various types of textual errors listed in Table 1. Similarly, while Mixtral was used to correct errors, many linguistic issues (such as grammatical and typological errors) were not included in the error rate quantification. As discussed previously, several specific error types, such as informal abbreviations and typographical mistakes, can be addressed under the broader category of spelling errors in the proposed error quantification metric. However, identifying specific issues within the text, especially those related to clinical language (eg, medical terms and jargon), requires further investigation. While spelling or typographical errors can be quantified at the token level, contextual errors, such as grammatical mistakes, are more challenging to assess and may require sentence-level evaluation. Conducting such analysis would require a high-quality dataset with detailed annotations for different types of errors. This analysis could be explored in future work without the use of machine learning models.

Second, we tested only a limited selection of feature representations and machine learning models, without conducting a systematic, in-depth analysis of the wide range of models available in the literature or performing comprehensive parameter tuning. Our analysis primarily focused on the ROC-AUC evaluation metric, which is widely used for assessing the performance of machine learning tasks. Comparative analysis of machine learning models relied mainly on the ROC-AUC metric. However, other metrics, such as recall, precision, and *F*_1_-score, are considered when true positives and false positives hold more significance. Since this study focuses on the impact of textual data quality on machine learning models, we did not explore the selection of models specifically optimized for tolerance to false positives while preserving true positives and negatives.

Lastly, we selected LLMs based on their computational feasibility and open-source availability, with only a limited comparative analysis between different models. Extending this study in the future to comprehensively and quantitatively evaluate various LLMs for data correction could provide valuable insights for the community in identifying models that offer the best performance in improving textual data quality across different types of errors.

### Conclusion

In this study, we evaluated the impact of low-quality textual data on feature representation and machine learning models. Unlike structured data, assessing the quality of textual data presents significant challenges due to the complex nature of many errors, such as grammatical, typographical, and missing-white space mistakes. To address this, we proposed a rudimentary error evaluation metric focused on spelling and missing-white space errors to assess the quality of two datasets: the real-world AACH dataset and the open-source MIMIC dataset. We systematically introduced errors into the MIMIC dataset to analyze their impact on feature representations and machine learning models. Additionally, we leveraged LLMs, particularly Mixtral, for identifying, correcting, and corrupting the textual data. By comparing the performance of models on datasets with varying error rates (evaluated in terms of ROC-AUC), we observed that models were relatively tolerant to smaller error rates (<10%) but their performance significantly deteriorated at higher error rates (≥10%). In the real-world AACH dataset, we measured an error rate of approximately 8%, which did not result in a major decline in predictive performance. Therefore, for datasets with higher error rates, corrective measures are essential to ensure the reliability of machine learning models.

The study also revealed that traditional TF-IDF techniques outperformed embedding models for medical predictive tasks, underscoring the importance of selecting feature representations tailored to the problem domain. Similarly, the choice of machine learning models is dataset-dependent. In this study, logistic regression yielded better results for the MIMIC dataset, while tree-based ensemble methods performed better for the AACH dataset.

## Appendix

Multimedia Appendix 1 Additional tables and figures.

## Abbreviations

AACH: Australian Aged Care Home
BERT: bidirectional encoder representations from transformers
EHR: electronic health records
IP: intellectual property
LLM: large language model
MLP: Multilayer Perceptron
NLP: natural language processing
ROC-AUC: area under the receiver operating curve
TF-IDF: term frequency–inverted document frequency

## Data Availability

The MIMIC-III dataset analyzed during this study is available at [71]. The AACH dataset analyzed during this study is not publicly available as the data was acquired from as the data was acquired from an aged care facility that did not provide consent for their data to be shared, but are available from Sarah Evans (aged.disability@health.telstra.com) on reasonable request.

## References

[1] Esteva A, Robicquet A, Ramsundar B, Kuleshov V, DePristo M, Chou K, Cui C, Corrado G, Thrun S, Dean J (2019). A guide to deep learning in healthcare. Nat Med.

[2] Jensen PB, Jensen LJ, Brunak S (2012). Mining electronic health records: towards better research applications and clinical care. Nat Rev Genet.

[3] Sarwar T, Seifollahi S, Chan J, Zhang X, Aksakalli V, Hudson I, Verspoor K, Cavedon L (2022). The secondary use of electronic health records for data mining: data characteristics and challenges. ACM Comput Surv.

[4] Yadav P, Steinbach M, Kumar V, Simon G (2018). Mining electronic health records (EHRs). ACM Comput Surv.

[5] Mahendra M, Luo Y, Mills H, Schenk G, Butte AJ, Dudley RA (2021). Impact of different approaches to preparing notes for analysis with natural language processing on the performance of prediction models in intensive care. Crit Care Explor.

[6] Luque C, Luna JM, Luque M, Ventura S (2019). An advanced review on text mining in medicine. WIREs Data Mining Knowl Discov.

[7] Zeng Z, Deng Y, Li X, Naumann T, Luo Y (2019). Natural language processing for EHR-Based computational phenotyping. IEEE/ACM Trans Comput Biol Bioinform.

[8] Spasic I, Nenadic G (2020). Clinical text data in machine learning: Systematic review. JMIR Med Inform.

[9] Yogarajan V, Montiel J, Smith T, Tucker A, Henriques Abreu P, Cardoso J, Pereira Rodrigues P, Riaño P (2021). Transformers for multi-label classification of medical text: an empirical comparison. Artificial Intelligence in Medicine (AIME 2021). Lecture Notes in Computer Science, vol 12721.

[10] Yang X, He X, Zhang H, Ma Y, Bian J, Wu Y (2020). Measurement of semantic textual similarity in clinical texts: comparison of transformer-based models. JMIR Med Inform.

[11] Gao S, Alawad M, Young MT, Gounley J, Schaefferkoetter N, Yoon HJ, Wu X, Durbin EB, Doherty J, Stroup A, Coyle L, Tourassi G (2021). Limitations of transformers on clinical text classification. IEEE J Biomed Health Inform.

[12] Lyu W, Dong X, Wong R, Zheng S, Abell-Hart K, Wang F, Chen C (2023). A multimodal transformer: fusing clinical notes with structured EHR data for interpretable in-hospital mortality prediction. AMIA Annu Symp Proc.

[13] Vaswani A, Shazeer N, Parmar N, Uszkoreit J, Jones L, Gomez AN, Kaiser Ł, Polosukhin I (2017). Attention is all you need.

[14] Zhao WX, Zhou K, Li J, Tang T, Wang X, Hou Y, Min Y, Zhang B, Zhang J, Dong Z, Du Y, Yang C, Chen Y, Chen Z, Jiang J, Ren R, Li Y, Tang X, Liu Z, Liu P, Nie J-Y, Wena J-R (2025). A survey of large language models. ArXiv. Preprint posted online on October 13, 2024.

[15] Chang Y, Wang X, Wang J, Wu Y, Yang L, Zhu K, Chen H, Yi X, Wang C, Wang Y, Ye W, Zhang Y, Chang Y, Yu PS, Yang Q, Xie X (2024). A survey on evaluation of large language models. ACM Trans Intell Syst Technol.

[16] Hadi MU, Tashi QA, Qureshi R, Shah A, Muneer A, Irfan M, Zafar A, Shaikh MB, Akhtar N, Hassan SN, Shoman M, Wu J, Mirjalili S, Shah M A survey on large language models: Applications, challenges, limitations, and practical usage. TechRxiv.

[17] Minaee S, Mikolov T, Nikzad N, Chenaghlu M, Socher R, Amatriain X, Gao J (2024). Large language models: a survey. ArXiv. Preprint posted online on February 9, 2024.

[18] Harrer S (2023). Attention is not all you need: the complicated case of ethically using large language models in healthcare and medicine. EBioMedicine.

[19] Lee J, Yoon W, Kim S, Kim D, Kim S, So CH, Kang J (2020). BioBERT: a pre-trained biomedical language representation model for biomedical text mining. Bioinformatics.

[20] Huang K, Altosaar J, Ranganath R (2020). ClinicalBERT: modeling clinical notes and predicting hospital readmission. ArXiv. Preprint posted online on November 29, 2020.

[21] Gu Y, Tinn R, Cheng H, Lucas M, Usuyama N, Liu X, Naumann T, Gao J, Poon H (2021). Domain-specific language model pretraining for biomedical natural language processing. ACM Trans Comput Healthcare.

[22] Gururangan S, Marasović A, Swayamdipta S, Lo K, Beltagy L, Downey D, Smith NA (2020). Don't stop pretraining: adapt language models to domains and tasks.

[23] Beltagy L, Lo K, Cohan A (2019). SciBERT: a pretrained language model for scientific text.

[24] Jin Q, Kim W, Chen Q, Comeau DC, Yeganova L, Wilbur WJ, Lu Z (2023). MedCPT: contrastive pre-trained transformers with large-scale PubMed search logs for zero-shot biomedical information retrieval. Bioinformatics.

[25] Yang X, Chen A, PourNejatian N, Shin HC, Smith KE, Parisien C, Compas C, Martin C, Costa AB, Flores MG, Zhang Y, Magoc T, Harle CA, Lipori G, Mitchell DA, Hogan WR, Shenkman EA, Bian J, Wu Y (2022). A large language model for electronic health records. NPJ Digit Med.

[26] Johnson AE, Pollard TJ, Shen L, Lehman LH, Feng M, Ghassemi M, Moody B, Szolovits P, Celi LA, Mark RG (2016). MIMIC-III, a freely accessible critical care database. Sci Data.

[27] Stubbs A, Uzuner Ö (2015). Annotating longitudinal clinical narratives for de-identification: The 2014 i2b2/UTHealth corpus. J Biomed Inform.

[28] Stubbs A, Kotfila C, Uzuner Ö (2015). Automated systems for the de-identification of longitudinal clinical narratives: overview of 2014 i2b2/UTHealth shared task Track 1. J Biomed Inform.

[29] Jain A, Patel H, Nagalapatti L, Gupta N, Mehta S, Guttula S, Mujumdar S, Afzal S, Mittal RS, Munigala V (2020). Overview and importance of data quality for machine learning tasks.

[30] Gupta N, Patel H, Afzal S, Panwar N, Mittal RS, Guttula S, Jain A, Nagalapatti L, Mehta S, Hans S, Lohia P, Aggarwal A, Saha D (2021). Data quality toolkit: automatic assessment of data quality and remediation for machine learning datasets. ArXiv. Preprint posted online on September 5, 2021.

[31] Polyzotis N, Zinkevich M, Roy S, Breck E, Whang S (2019). Data validation for machine learning. Proc Mach Learn Res.

[32] Chen H, Chen J, Ding J (2021). Data evaluation and enhancement for quality improvement of machine learning. IEEE Trans Rel.

[33] Gudivada VN, Apon A, Ding J (2017). Data quality considerations for big data and machine learning: going beyond data cleaning and transformations. Int J Adv Softw.

[34] Afzal S, Rajmohan C, Kesarwani M, Mehta S, Patela H (2021). Data Readiness Report.

[35] McGilvray D (2021). Executing Data Quality Projects Ten Steps to Quality Data and Trusted Information. Second Edition.

[36] Chandran DR, Gupta V (2022). A short review of the literature on automatic data quality. J Comput Commun.

[37] Chen H, Cao G, Chen J, Ding J, Zhu X, Qin B, Zhu X, Liu M, Qian L (2019). A practical framework for evaluating the quality of knowledge graph. Knowledge Graph and Semantic Computing: Knowledge Computing and Language Understanding (CCKS 2019).

[38] Razzaghi H, Greenberg J, Bailey LC (2022). Developing a systematic approach to assessing data quality in secondary use of clinical data based on intended use. Learn Health Syst.

[39] Weiskopf NG, Hripcsak G, Swaminathan S, Weng C (2013). Defining and measuring completeness of electronic health records for secondary use. J Biomed Inform.

[40] Chan KS, Fowles JB, Weiner JP (2010). Review: electronic health records and the reliability and validity of quality measures: a review of the literature. Med Care Res Rev.

[41] Kiefer C, Meyer H (2019). Quality indicators for text data. BTW 2019 — Workshopband.

[42] Larson S, Mahendran A, Lee A, Kummerfeld JK, Hill P, Laurenzano MA, Hauswald J, Tang L, Mars J (2019). Outlier detection for improved data quality and diversity in dialog systems.

[43] Peinelt N, Liakata M, Nguyen D (2019). Aiming beyond the obvious: identifying non-obvious cases in semantic similarity datasets.

[44] Collins E, Rozanov N, Zhang B (2018). Evolutionary data measures: understanding the difficulty of text classification tasks.

[45] Jiang S, Ren P, Monz C, de Rijke M (2019). Improving neural response diversity with frequency-aware cross-entropy loss.

[46] Northcutt C, Jiang L, Chuang I (2021). Confident learning: estimating uncertainty in dataset labels. J Artif Intell Res.

[47] Dickinson M, Meurers WD (2003). Detecting errors in part-of-speech annotation.

[48] Eskin E (2000). Detecting errors within a corpus using anomaly detection. https://aclanthology.org/A00-2020.pdf.

[49] Rehbein I, Ruppenhofer J (2017). Detecting annotation noise in automatically labelled data. https://aclanthology.org/P17-1107/.

[50] Gupta N, Mujumdar S, Patel H, Masuda S, Panwar N, Bandyopadhyay S, Mehta S, Guttula S, Afzal A, Mittal RS, Munigala C (2021). Data quality for machine learning tasks.

[51] Swayamdipta S, Schwartz R, Lourie N, Wang Y, Hajishirzi H, Smith NA, Choi Y (2020). Dataset cartography: mapping and diagnosing datasets with training dynamics. https://aclanthology.org/2020.emnlp-main.746/.

[52] Ribeiro MT, Wu T, Guestrin C, Singh S (2020). Beyond accuracy: behavioral testing of NLP models with checklist. https://aclanthology.org/2020.acl-main.442/.

[53] Colavito G, Lanubile F, Novielli N, Quaranta L (2024). Impact of data quality for automatic issue classification using pre-trained language models. J Syst Softw.

[54] AutoCorrect.

[55] Pyaspeller.

[56] Grammar Check.

[57] Wang Y, Wang Y, Dang K, Liu J, Liu Z (2021). A comprehensive survey of grammatical error correction. ACM Trans Intell Syst Technol.

[58] Ng HT, Wu SM, Briscoe T, Hadiwinoto C, Susanto RH, Bryant C (2014). The CoNLL-2014 shared task on grammatical error correction. https://aclanthology.org/W14-1701/.

[59] Bryant C, Yuan Z, Qorib MR, Cao H, Ng HT, Briscoe T (2023). Grammatical error correction: a survey of the state of the art. Comput Linguist.

[60] Rangapur A, Rangapur A (2024). The battle of LLMs: A comparative study in conversational QA tasks. ArXiv. Preprint posted online on October 13, 2024.

[61] Widyasari R, Lo D, Liao L (2024). Beyond ChatGPT: enhancing software quality assurance tasks with diverse LLMs and validation techniques. ArXiv. Preprint posted online on September 2, 2024.

[62] Jiang AQ, Sablayrolles A, Roux A, Mensch A, Savary B, Bamford C, Chaplot DS, de las Casas D, Hanna EB, Bressand F, Lengyel G, Bour G, Lample G, Lavaud LR, Saulnier L, Lachaux M-A, Stock P, Subramanian S, Yang S, Antoniak S, Le Scao T, Gervet T, Lavril T, Wang T, Lacroix T, El Sayed W (2024). Mixtral of experts. ArXiv. Preprint posted online on January 8, 2024.

[63] Mahbub M, Srinivasan S, Danciu I, Peluso A, Begoli E, Tamang S, Peterson GD (2022). PLoS One.

[64] Mikolov T, Sutskever I, Chen K, Corrado G, Dean J (2013). Distributed representations of words and phrases and their compositionality. https://papers.nips.cc/paper_files/paper/2013/hash/9aa42b31882ec039965f3c4923ce901b-Abstract.html.

[65] Devlin J, Chang M-W, Lee K, Toutanova K (2019). BERT: Pre-training of deep bidirectional transformers for language understanding. https://aclanthology.org/N19-1423/.

[66] Alsentzer E, Murphy J, Boag W, Weng W-H, Jindi D, Naumann T, McDermott M (2019). Publicly available clinical BERT embeddings. https://aclanthology.org/W19-1909/.

[67] Beltagy I, Peters ME, Cohan A (2020). Longformer: the long-document transformer. ArXiv. Preprint posted online on December 2, 2020.

[68] Sounack T, Davis J, Durieux B, Chaffin A, Pollard TJ, Lehman E, Johnson AEW, McDermott M, Naumann T, Lindvall C (2025). BioClinical ModernBERT: A state-of-the-art long-context encoder for biomedical and clinical NLP. ArXiv. Preprint posted online on June 18, 2025.

[69] Wang L, Yang N, Huang X, Yang L, Majumder R, Wei F (2023). Improving text embeddings with large language models. ArXiv. Preprint posted online on January 1, 2024.

[70] Arora A, Liang Y, Ma T (2017). A simple but tough-to-beat baseline for sentence embeddings. ICLR.

[71] Johnson A, Pollard T, Mark R (2016). MIMIC-III Clinical Database.

