# Supplementary material for: Assessing the Impact of the Quality of Textual Data on Feature Representation and Machine Learning Models: Quantitative Study Using Large Language Models
Source: J Med Internet Res. 2025 Dec 30;27:e73325. doi: 10.2196/73325 (PMC12811037; doi:10.2196/73325)
Supplement: Multimedia Appendix 1 [file jmir_v27i1e73325_app1.docx]

**Supplementary Data**

**Table S1.** Mixtral model settings used in this study.

| **Hyper-parameters** | For text generation, we used the Hugging Face transformers library with the Mixtral-8x7B-Instruct-v0.1 model. Unless otherwise specified, we employed **greedy decoding** by setting do_sample=False, which disables sampling and results in deterministic generation. In this mode, parameters such as temperature, top_k, and top_p are ignored, as the model always selects the token with the highest probability at each step. Default parameters used by the library were used for the experiments. |
| --- | --- |
| **Prompt for extracting erroneous tokens** | Prompt = """do any tokens in the following list have a spelling mistake, missing space or punctuation errors? generate a table with two columns, the first column represents only the tokens containing errors and the other column represents the corrected token: {list_of_tokens}""" |
| **Prompt for text correction** | Prompt = """ do any tokens in the following list have a spelling mistake, missing space or punctuation errors? generate a table with two columns, the first column represents only the tokens containing errors and the other column represents the corrected token: {list_of_tokens}""" |
| **Sample Codes** | <https://github.com/sarwart/mlModels> |

**Table S2**. Sample queries demonstrating the extraction of tokens with errors using Mixtral. The blue text represents the base query used for all data instances, while the red text was replaced for different data instances.

| **Ind.** | **Example Queries** | **Mixtral Outcome** |
| --- | --- | --- |
| 1 | do any tokens in the following list have a spelling mistake, missing space or punctuation errors? generate a table with two columns, the first column represents only the tokens containing errors and the other column represents the corrected token: [‘The’, ‘resident’, ‘didnot', ‘eat’, ‘much’, ‘for’, ‘dinner’, ‘she’, ‘had’, ‘a’, ‘cup’, ‘tea’, ‘few’, ‘spoonful’, ‘of’, ‘soup’ , ‘and’, ‘main’, ‘meal.’] | 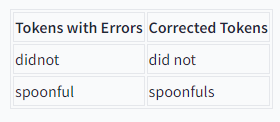 |
| 2 | do any tokens in the following list have a spelling mistake, missing space or punctuation errors? generate a table with two columns, the first column represents only the tokens containing errors and the other column represents the corrected token: [‘XYZ’, ‘is’, ‘also’, ‘walked’, ‘to’, ‘crafts,and’, ‘atttend’, ‘as’, ‘many’, ‘group’, ‘activities’, ‘as’, ‘she’, ‘can,too,decreased’, ‘isolation.’] | 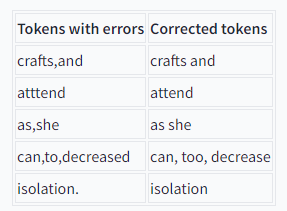 |

**Table S3**. Sample queries demonstrating the data correction using Mixtral. The blue text represents the base query used for all data instances, while the red text was replaced for different data instances. Text linked with the identifiable information has been removed.

| **Ind.** | **Example Queries** | **Mixtral Outcome** |
| --- | --- | --- |
| 1. | Strictly proofread and correct any spelling mistakes in the following sentences. Only produce a single corrected text and do not provide any explanation. Return 'nil' if there is no mistake: “Resident buzzed atTIMEhrs onDATE,her legs felt like they were burning n she was in pain” | "The resident buzzed at TIME hours on DATE; her legs felt like they were burning, and she was in pain." |
| 2. | Strictly proofread and correct any spelling mistakes in the following sentences. Only produce a single corrected text and do not provide any explanation. Return 'nil' if there is no mistake: “Resident was sleeping on round check,repositioned by2 x staff fluids given. nil problems settled ator” | "The resident was sleeping during routine check; repositioned by two staff members. No fluid issues were settled or ator." |

**Table S4**. Sample queries demonstrating the data correction using Mixtral on token level. The blue text represents the base query used for all data instances, while the red text was replaced for different data instances. Text linked with the identifiable information has been removed.

| **Ind.** | **Example Queries** | **Mixtral Outcome** |
| --- | --- | --- |
| 1. | Correct any spelling mistakes in the following list. Only generate a list containing all the provided tokens along with the ones that have been corrected do not provide any explanation: ["Resident", "buzzed", "atTIMEhrs", "onDATE,her", "legs", "felt", "like", "they", "were", "burning", "n", "she", "was", "in", "pain”] | ["Resident", "buzzed", "at TIME hours", "on DATE", "her legs", "felt like", "they were burning", "she was in", "pain"] |
| 2. | Correct any spelling mistakes in the following list. Only generate a list containing all the provided tokens along with the ones that have been corrected do not provide any explanation: ["Resident", "was", "sleeping", "on", "round", "check,repositioned", "by2", "x", "staff", "fluids", "given.", "nil", "problems", "settled", "ator"] | ["Resident", "was", "sleeping", "on", "the", "round", "check, repositioned", "by", "staff", "fluids", "given.", "nil", "problems", "were", "settled", "after", "the", "ator"] |

**Table S5.** Regular expression-based tokenizer statistics for different datasets and prediction tasks. This tokenization approach was used for TF-IDF and word2vec approaches.

| **Dataset** | **Prediction Task** | **Experiment Condition** | **Unique tokens** | **Avg length (std)** | **Max length** | **Min length** |
| --- | --- | --- | --- | --- | --- | --- |
| MIMIC III | Mortality | Original notes (no error) | 129,069 | 1,641.28 (2,372.09) | 29,715 | 4 |
|  |  | 5% error | 494,049 | 1,567.90 (2,278.57) | 28,300 | 4 |
|  |  | 10% error | 740,008 | 1,531.81 (2,229.66) | 27,693 | 4 |
|  |  | 15% error | 973,248 | 1,498.84 (2,188.69) | 27,478 | 3 |
|  |  | 20% error | 1,219,423 | 1,468.29 (2,152.54) | 27,227 | 3 |
| Aged Care Home | Depression | 30-days tracking - original | 59,535 | 2,578.13 (2,322.06) | 18,616 | 7 |
|  |  | 30-days tracking - corrected | 38,307 | 2,721.01 (2,446.99) | 19,373 | 8 |
|  |  | 60-days tracking - original | 74,904 | 4,037.24 (3,623.64) | 30,432 | 7 |
|  |  | 60-days tracking - corrected | 46,090 | 4,258.67 (3,817.11) | 32,178 | 8 |
|  |  | 90-days tracking - original | 84,023 | 5,070.59 (4,531.26) | 34,089 | 7 |
|  |  | 90-days tracking - corrected | 50,675 | 5,350.08 (4,778.42) | 36,141 | 8 |
|  | First Fall | 30-days tracking - original | 48,743 | 1,922.27 (1,522.90) | 19,844 | 10 |
|  |  | 30-days tracking - corrected | 32,769 | 2,029.64 (1,603.87) | 20,287 | 11 |
|  |  | 60-days tracking - original | 60,443 | 3,110.62 (2,618.96) | 21,905 | 12 |
|  |  | 60-days tracking - corrected | 38,513 | 3,280.91 (2,757.69) | 22,827 | 12 |
|  |  | 90-days tracking - original | 66,593 | 4,117.92 (3,408.97) | 28,333 | 12 |
|  |  | 90-days tracking - corrected | 41,440 | 4,341.63 (3,589.90) | 29,486 | 12 |
|  | Fall with Fall History | 30-days tracking - original | 63,814 | 1,760.24 (1,422.99) | 19,844 | 10 |
|  |  | 30-days tracking - corrected | 40,335 | 1,858.47 (1,499.21) | 20,287 | 11 |
|  |  | 60-days tracking - original | 81,808 | 3,001.53 (2,356.72) | 21,456 | 23 |
|  |  | 60-days tracking - corrected | 48,875 | 3,163.77 (2,477.03) | 22,672 | 23 |
|  |  | 90-days tracking - original | 91,450 | 4,140.68 (3,099.86) | 27,850 | 18 |
|  |  | 90-days tracking - corrected | 53,532 | 4,365.00 (3,262.56) | 29,385 | 20 |

**Table S6.** BERT tokenizer statistics for different datasets and prediction tasks.

| **Dataset** | **Prediction Task** | **Experiment Condition** | **Unique tokens** | **Avg length (std)** | **Max length** | **Min length** |
| --- | --- | --- | --- | --- | --- | --- |
| MIMIC III | Mortality | Original notes (no error) | 15,465 | 3,955.69 (5,634.82) | 66,833 | 9 |
|  |  | 5% error | 15,719 | 3,583.78 (5,074.80) | 61,179 | 9 |
|  |  | 10% error | 15,849 | 3,634.20 (5,153.00) | 61,934 | 9 |
|  |  | 15% error | 15,888 | 3,687.11 (5,241.47) | 63,952 | 8 |
|  |  | 20% error | 15,942 | 3,744.27 (5,341.49) | 66,215 | 10 |
| Aged Care Home | Depression | 30-days tracking - original | 14,236 | 3,618.31 (3,245.75) | 26,398 | 11 |
|  |  | 30-days tracking - corrected | 14,495 | 3,760.94 (3,356.32) | 27,327 | 11 |
|  |  | 60-days tracking - original | 14,924 | 5,658.85 (5,066.26) | 42,983 | 11 |
|  |  | 60-days tracking - corrected | 15,205 | 5,882.29 (5,243.26) | 44,600 | 11 |
|  |  | 90-days tracking - original | 15,227 | 7,110.86 (6,373.50) | 48,197 | 11 |
|  |  | 90-days tracking - corrected | 15,554 | 7,396.76 (6,608.38) | 50,184 | 11 |
|  | First Fall | 30-days tracking - original | 13,675 | 2,628.43 (2,063.69) | 26,407 | 16 |
|  |  | 30-days tracking - corrected | 13,991 | 2,747.68 (2,139.08) | 26,235 | 17 |
|  |  | 60-days tracking - original | 14,293 | 4,229.56 (3,528.94) | 30,057 | 16 |
|  |  | 60-days tracking - corrected | 14,638 | 4,418.15 (3,666.33) | 31,065 | 17 |
|  |  | 90-days tracking - original | 14,530 | 5,591.90 (4,595.62) | 38,343 | 16 |
|  |  | 90-days tracking - corrected | 14,879 | 5,842.49 (4,779.39) | 39,828 | 17 |
|  | Fall with Fall History | 30-days tracking - original | 14,423 | 2,399.27 (1,929.27) | 26,407 | 16 |
|  |  | 30-days tracking - corrected | 14,774 | 2,509.76 (2,001.56) | 26,235 | 17 |
|  |  | 60-days tracking - original | 15,061 | 4,073.35 (3,188.23) | 30,057 | 35 |
|  |  | 60-days tracking - corrected | 15,423 | 4,257.21 (3,305.07) | 31,065 | 34 |
|  |  | 90-days tracking - original | 15,347 | 5,618.83 (4,190.68) | 38,343 | 27 |
|  |  | 90-days tracking - corrected | 15,770 | 5,872.59 (4,353.04) | 39,828 | 29 |

**Table S7.** Number of features for different NLP feature representation models for mortality prediction task for the MIMIC dataset.

| **Feature Representation** | **Number of features (dimensionality of input vector)** | | | | |
| --- | --- | --- | --- | --- | --- |
|  | **Original** | **5% error rate** | **10% error rate** | **15% error rate** | **20% error rate** |
| TF-IDF (top) | 5,000 | | | | |
| TF-IDF (min) | 32,804 | 84,962 | 119,875 | 148,736 | 175,915 |
| TF-TDF (raw) | 128,921 | 493,901 | 739,859 | 973,099 | 1,219,274 |
| word2vec | 300 | | | | |
| BERT | 786 | | | | |

**Table S8.** Number of features for different NLP feature representation models for the deterioration prediction tasks in the AACH setting. TF-IDF (raw) represents the case when filtering is not applied to TF-IDF features

| **Deterioration Prediction Tasks** | **Feature Representation** | **Number of features (dimensionality of input vector)** | | | | | |
| --- | --- | --- | --- | --- | --- | --- | --- |
|  |  | **30-day tracking** | | **60-day tracking** | | **90-day tracking** | |
|  |  | **Original** | **Corrected** | **Original** | **Corrected** | **Original** | **Corrected** |
| Depression | TF-IDF (min) | 14,626 | 13,208 | 18,007 | 15,631 | 20,042 | 17,003 |
|  | TF-IDF (raw) | 59,388 | 38,156 | 74,756 | 45,939 | 83,873 | 50,523 |
| First Fall | TF-IDF (min) | 14,265 | 12,807 | 16,040 | 14,084 | 16,817 | 14,610 |
|  | TF-IDF (raw) | 48,606 | 32,621 | 60,304 | 38,364 | 66,453 | 41,290 |
| Fall with history | TF-IDF (min) | 17,380 | 15,009 | 21,329 | 17,594 | 23,150 | 18,740 |
|  | TF-IDF (raw) | 63,675 | 40,185 | 81,667 | 48,725 | 91,310 | 53,381 |
| Common across all the tasks | TF-IDF (top) | 5,000 | | | | | |
|  | word2vec | 300 | | | | | |
|  | BERT | 768 | | | | | |

**
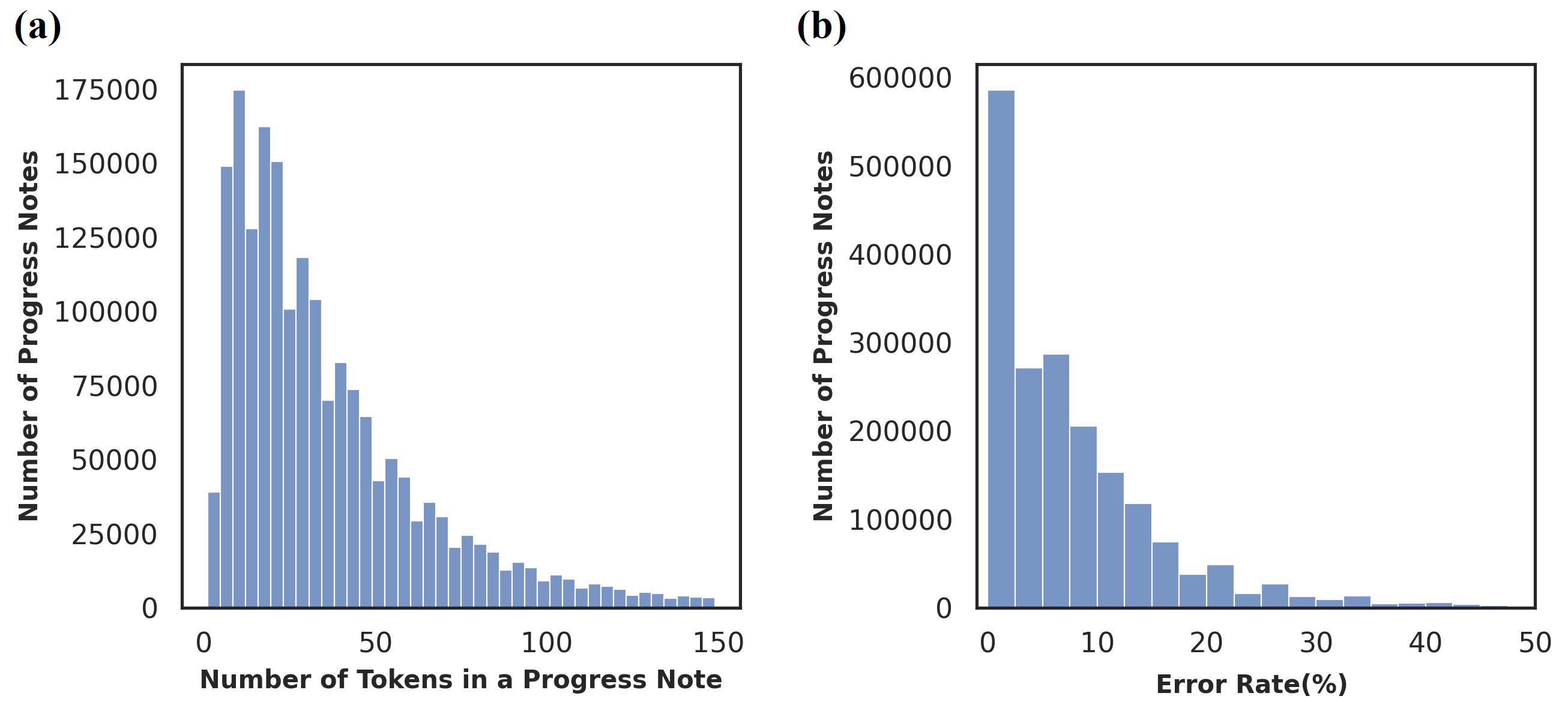
**

**Figure S1.** (a) The histogram demonstrating the number of tokens (length) in progress notes. AACH dataset consisted of 1,903,936 progress notes. A threshold of <150 tokens was applied for clarity of the figure. It should be noted that 40,032 (2.1%) progress notes have a length greater than 150 tokens. (b) The extent of errors found in the AACH dataset.

**Table S9.** The performance of machine learning models under varying hyperparameters on the MIMIC dataset (**original dataset**). The default hyperparameters from the scikit-learn library are used unless specifically stated under 'Parameters.' The comparatively best ROC-AUC value for each machine learning model is highlighted in blue. For brevity, the results corresponding to only **TF-IDF** feature representation are mentioned here.

| **Machine Learning Model** | **Parameters** | **ROC - AUC** | **Precision** | | **Recall** | | **F1-Score** | |
| --- | --- | --- | --- | --- | --- | --- | --- | --- |
|  |  |  | **Class label - 0** | **Class label - 1** | **Class label - 0** | **Class label - 1** | **Class label - 0** | **Class label - 1** |
| Logistic Regression | C=0.5, class_weight = 'balanced' | 0.860 | 0.974 | 0.242 | 0.794 | 0.757 | 0.875 | 0.367 |
| Random Forest Classifier | default | 0.802 | 0.920 | 1.000 | 1.000 | 0.001 | 0.958 | 0.002 |
|  | n_estimators = 300 | 0.822 | 0.920 | 0.000 | 1.000 | 0.000 | 0.958 | 0.000 |
|  | n_estimators = 500 | 0.825 | 0.920 | 0.000 | 1.000 | 0.000 | 0.958 | 0.000 |
|  | max_depth=3 | 0.743 | 0.920 | 0.000 | 1.000 | 0.000 | 0.958 | 0.000 |
|  | max_depth=3, n_estimators=300 | 0.756 | 0.920 | 0.000 | 1.000 | 0.000 | 0.958 | 0.000 |
|  | max_depth=3, n_estimators=500 | 0.756 | 0.920 | 0.000 | 1.000 | 0.000 | 0.958 | 0.000 |
| XGB Classifier | default | 0.807 | 0.950 | 0.262 | 0.887 | 0.462 | 0.917 | 0.334 |
|  | n_estimators=100 | 0.807 | 0.950 | 0.262 | 0.887 | 0.462 | 0.917 | 0.334 |
|  | n_estimators=300 | 0.803 | 0.938 | 0.334 | 0.951 | 0.282 | 0.945 | 0.306 |
|  | n_estimators=500 | 0.808 | 0.935 | 0.396 | 0.971 | 0.220 | 0.952 | 0.283 |
|  | max_depth=3 | 0.836 | 0.969 | 0.230 | 0.796 | 0.703 | 0.874 | 0.347 |
|  | max_depth=3, n_estimators=100 | 0.836 | 0.969 | 0.230 | 0.796 | 0.703 | 0.874 | 0.347 |
|  | max_depth=3, n_estimators=300 | 0.826 | 0.958 | 0.260 | 0.861 | 0.563 | 0.907 | 0.356 |
|  | max_depth=3, n_estimators=500 | 0.818 | 0.951 | 0.280 | 0.895 | 0.471 | 0.922 | 0.351 |
| MLP Classifier | early_stopping=True, max_iter=300 | 0.851 | 0.934 | 0.519 | 0.984 | 0.196 | 0.958 | 0.285 |
|  | early_stopping=True, hidden_layer_sizes=( 100,50,25), max_iter=300 | 0.856 | 0.938 | 0.511 | 0.978 | 0.261 | 0.958 | 0.345 |
| SVC | cache_size=10000, class_weight= 'balanced', kernel='linear', probability=True | 0.838 | 0.966 | 0.241 | 0.816 | 0.673 | 0.885 | 0.355 |
|  | C=0.5, cache_size=10000, class_weight= 'balanced', kernel='linear', probability=True | 0.846 | 0.970 | 0.232 | 0.792 | 0.721 | 0.872 | 0.351 |
|  | cache_size=10000, class_weight= 'balanced', kernel=’rbf’, probability=True | 0.854 | 0.948 | 0.414 | 0.951 | 0.398 | 0.949 | 0.406 |
|  | C=0.5, cache_size=10000, class_weight=' balanced', kernel=’rbf’, probability=True | 0.857 | 0.960 | 0.320 | 0.895 | 0.571 | 0.926 | 0.410 |

**Table S10.** The performance of machine learning models under varying hyperparameters on the MIMIC dataset with **5% error-rate**. The default hyperparameters from the scikit-learn library are used unless specifically stated under 'Parameters.' The comparatively best ROC-AUC value for each machine learning model is highlighted in blue. For brevity, the results corresponding to only **TF-IDF** feature representation are mentioned here.

| **Machine Learning Model** | **Parameters** | **ROC - AUC** | **Precision** | | **Recall** | | **F1-Score** | |
| --- | --- | --- | --- | --- | --- | --- | --- | --- |
|  |  |  | **Class label - 0** | **Class label - 1** | **Class label - 0** | **Class label - 1** | **Class label - 0** | **Class label - 1** |
| Logistic Regression | C=0.5, class_weight = 'balanced' | 0.858 | 0.972 | 0.241 | 0.799 | 0.736 | 0.877 | 0.363 |
| Random Forest Classifier | default | 0.795 | 0.920 | 0.000 | 1.000 | 0.000 | 0.958 | 0.000 |
|  | n_estimators=300 | 0.813 | 0.920 | 0.000 | 1.000 | 0.000 | 0.958 | 0.000 |
|  | n_estimators=500 | 0.816 | 0.920 | 0.000 | 1.000 | 0.000 | 0.958 | 0.000 |
|  | max_depth=3 | 0.737 | 0.920 | 0.000 | 1.000 | 0.000 | 0.958 | 0.000 |
|  | max_depth=3, n_estimators=300 | 0.744 | 0.920 | 0.000 | 1.000 | 0.000 | 0.958 | 0.000 |
|  | max_depth=3, n_estimators=500 | 0.748 | 0.920 | 0.000 | 1.000 | 0.000 | 0.958 | 0.000 |
| XGB Classifier | default | 0.805 | 0.950 | 0.252 | 0.880 | 0.466 | 0.914 | 0.327 |
|  | n_estimators=100 | 0.805 | 0.950 | 0.252 | 0.880 | 0.466 | 0.914 | 0.327 |
|  | n_estimators=300 | 0.802 | 0.940 | 0.324 | 0.945 | 0.302 | 0.943 | 0.313 |
|  | n_estimators=500 | 0.804 | 0.936 | 0.390 | 0.968 | 0.234 | 0.952 | 0.292 |
|  | max_depth=3 | 0.831 | 0.967 | 0.216 | 0.781 | 0.696 | 0.864 | 0.330 |
|  | max_depth=3, n_estimators=100 | 0.831 | 0.967 | 0.216 | 0.781 | 0.696 | 0.864 | 0.330 |
|  | max_depth=3, n_estimators=300 | 0.824 | 0.958 | 0.257 | 0.858 | 0.565 | 0.905 | 0.354 |
|  | max_depth=3, n_estimators=500 | 0.815 | 0.951 | 0.275 | 0.895 | 0.459 | 0.922 | 0.344 |
| MLP Classifier | early_stopping=True, max_iter=300 | 0.853 | 0.932 | 0.561 | 0.988 | 0.170 | 0.959 | 0.261 |
|  | early_stopping=True, hidden_layer_sizes= (100,50,25), max_iter= 300 | 0.850 | 0.938 | 0.531 | 0.981 | 0.251 | 0.959 | 0.340 |
| SVC | cache_size=10000, class_weight= 'balanced', kernel= 'linear', probability= True | 0.835 | 0.963 | 0.259 | 0.843 | 0.631 | 0.899 | 0.367 |
|  | C=0.5, cache_size =10000, class_weight= 'balanced', kernel= 'linear', probability=True | 0.844 | 0.969 | 0.240 | 0.808 | 0.698 | 0.881 | 0.357 |
|  | cache_size= 10000, class_weight= 'balanced', kernel=’rbf’, probability= True | 0.853 | 0.945 | 0.446 | 0.962 | 0.356 | 0.953 | 0.396 |
|  | C=0.5, cache_size= 10000, class_weight= 'balanced', kernel=’rbf’, probability =True | 0.856 | 0.957 | 0.333 | 0.908 | 0.528 | 0.932 | 0.408 |

**Table S11.** The performance of machine learning models under varying hyperparameters on the MIMIC dataset with **10% error-rate**. The default hyperparameters from the scikit-learn library are used unless specifically stated under 'Parameters.' The comparatively best ROC-AUC value for each machine learning model is highlighted in blue. For brevity, the results corresponding to only **TF-IDF** feature representation are mentioned here.

| **Machine Learning Model** | **Parameters** | **ROC - AUC** | **Precision** | | **Recall** | | **F1-Score** | |
| --- | --- | --- | --- | --- | --- | --- | --- | --- |
|  |  |  | **Class label - 0** | **Class label - 1** | **Class label - 0** | **Class label - 1** | **Class label - 0** | **Class label - 1** |
| Logistic Regression | C=0.5, class_weight= 'balanced' | 0.856 | 0.971 | 0.243 | 0.803 | 0.728 | 0.879 | 0.364 |
| Random Forest Classifier | default | 0.793 | 0.920 | 0.333 | 1.000 | 0.001 | 0.958 | 0.002 |
|  | n_estimators=300 | 0.815 | 0.920 | 0.000 | 1.000 | 0.000 | 0.958 | 0.000 |
|  | n_estimators=500 | 0.821 | 0.920 | 0.000 | 1.000 | 0.000 | 0.958 | 0.000 |
|  | max_depth=3 | 0.740 | 0.920 | 0.000 | 1.000 | 0.000 | 0.958 | 0.000 |
|  | max_depth=3, n_estimators= 300 | 0.744 | 0.920 | 0.000 | 1.000 | 0.000 | 0.958 | 0.000 |
|  | max_depth=3, n_estimators= 500 | 0.751 | 0.920 | 0.000 | 1.000 | 0.000 | 0.958 | 0.000 |
| XGB Classifier | default | 0.811 | 0.953 | 0.258 | 0.876 | 0.498 | 0.912 | 0.340 |
|  | n_estimators=100 | 0.811 | 0.953 | 0.258 | 0.876 | 0.498 | 0.912 | 0.340 |
|  | n_estimators=300 | 0.805 | 0.941 | 0.333 | 0.945 | 0.317 | 0.943 | 0.325 |
|  | n_estimators=500 | 0.808 | 0.937 | 0.397 | 0.967 | 0.253 | 0.952 | 0.309 |
|  | max_depth=3 | 0.828 | 0.967 | 0.217 | 0.783 | 0.691 | 0.865 | 0.330 |
|  | max_depth=3, n_estimators= 100 | 0.828 | 0.967 | 0.217 | 0.783 | 0.691 | 0.865 | 0.330 |
|  | max_depth=3, n_estimators= 300 | 0.813 | 0.955 | 0.240 | 0.850 | 0.544 | 0.900 | 0.333 |
|  | max_depth=3, n_estimators= 500 | 0.812 | 0.951 | 0.268 | 0.889 | 0.468 | 0.919 | 0.341 |
| MLP Classifier | early_stopping=True, max_iter= 300 | 0.847 | 0.928 | 0.584 | 0.993 | 0.114 | 0.959 | 0.191 |
|  | early_stopping=True, hidden_layer_sizes= (100,50,25), max_iter= 300 | 0.845 | 0.920 | 0.000 | 1.000 | 0.000 | 0.958 | 0.000 |
| SVC | cache_size=10000, class_weight= 'balanced', kernel= 'linear', probability= True | 0.831 | 0.960 | 0.265 | 0.859 | 0.585 | 0.907 | 0.365 |
|  | C=0.5, cache_size=10000, class_weight= 'balanced', kernel= 'linear', probability=True | 0.842 | 0.966 | 0.244 | 0.819 | 0.670 | 0.887 | 0.357 |
|  | cache_size=10000, class_weight= 'balanced', kernel=’rbf’, probability= True | 0.851 | 0.944 | 0.480 | 0.969 | 0.333 | 0.956 | 0.393 |
|  | C=0.5, cache_size=10000, class_weight= 'balanced', kernel=’rbf’, probability= True | 0.854 | 0.955 | 0.344 | 0.917 | 0.503 | 0.935 | 0.409 |

**Table S12.** The performance of machine learning models under varying hyperparameters on the MIMIC dataset with **15% error-rate**. The default hyperparameters from the scikit-learn library are used unless specifically stated under 'Parameters.' The comparatively best value for each machine learning model is highlighted in blue. For brevity, the results corresponding to only TF-IDF feature representation are mentioned here.

| **Machine Learning Model** | **Parameters** | **ROC - AUC** | **Precision** | | **Recall** | | **F1-Score** | |
| --- | --- | --- | --- | --- | --- | --- | --- | --- |
|  |  |  | **Class label - 0** | **Class label - 1** | **Class label - 0** | **Class label - 1** | **Class label - 0** | **Class label - 1** |
| Logistic Regression | C=0.5, class_weight= 'balanced' | 0.856 | 0.971 | 0.246 | 0.808 | 0.720 | 0.882 | 0.367 |
| Random Forest Classifier | default | 0.788 | 0.920 | 0.000 | 1.000 | 0.000 | 0.958 | 0.000 |
|  | n_estimators=300 | 0.809 | 0.920 | 0.000 | 1.000 | 0.000 | 0.958 | 0.000 |
|  | n_estimators=500 | 0.818 | 0.920 | 0.000 | 1.000 | 0.000 | 0.958 | 0.000 |
|  | max_depth=3 | 0.746 | 0.920 | 0.000 | 1.000 | 0.000 | 0.958 | 0.000 |
|  | max_depth=3, n_estimators= 300 | 0.740 | 0.920 | 0.000 | 1.000 | 0.000 | 0.958 | 0.000 |
|  | max_depth=3, n_estimators= 500 | 0.743 | 0.920 | 0.000 | 1.000 | 0.000 | 0.958 | 0.000 |
| XGB Classifier | default | 0.809 | 0.950 | 0.252 | 0.878 | 0.471 | 0.913 | 0.328 |
|  | n_estimators=100 | 0.809 | 0.950 | 0.252 | 0.878 | 0.471 | 0.913 | 0.328 |
|  | n_estimators=300 | 0.804 | 0.939 | 0.322 | 0.945 | 0.299 | 0.942 | 0.310 |
|  | n_estimators=500 | 0.807 | 0.936 | 0.376 | 0.966 | 0.237 | 0.951 | 0.291 |
|  | max_depth=3 | 0.830 | 0.968 | 0.224 | 0.789 | 0.702 | 0.870 | 0.340 |
|  | max_depth=3, n_estimators= 100 | 0.830 | 0.968 | 0.224 | 0.789 | 0.702 | 0.870 | 0.340 |
|  | max_depth=3, n_estimators= 300 | 0.823 | 0.958 | 0.258 | 0.858 | 0.570 | 0.905 | 0.356 |
|  | max_depth=3, n_estimators= 500 | 0.816 | 0.951 | 0.280 | 0.896 | 0.466 | 0.922 | 0.350 |
| MLP Classifier | early_stopping=True, max_iter= 300 | 0.838 | 0.930 | 0.531 | 0.989 | 0.146 | 0.959 | 0.229 |
|  | early_stopping=True, hidden_layer_sizes= (100,50,25), max_iter= 300 | 0.829 | 0.930 | 0.506 | 0.988 | 0.147 | 0.958 | 0.227 |
| SVC | cache_size=10000, class_weight= 'balanced', kernel= 'linear', probability= True | 0.835 | 0.958 | 0.285 | 0.878 | 0.562 | 0.916 | 0.379 |
|  | C=0.5, cache_size=10000, class_weight= 'balanced', kernel= 'linear', probability= True | 0.844 | 0.967 | 0.260 | 0.834 | 0.672 | 0.895 | 0.375 |

**Table S13.** The performance of machine learning models under varying hyperparameters on the MIMIC dataset with **20% error-rate**. The default hyperparameters from the scikit-learn library are used unless specifically stated under 'Parameters.' The comparatively best ROC-AUC value for each machine learning model is highlighted in blue. For brevity, the results corresponding to only **TF-IDF** feature representation are mentioned here.

| **Machine Learning Model** | **Parameters** | **ROC - AUC** | **Precision** | | **Recall** | | **F1-Score** | |
| --- | --- | --- | --- | --- | --- | --- | --- | --- |
|  |  |  | **Class label - 0** | **Class label - 1** | **Class label - 0** | **Class label - 1** | **Class label - 0** | **Class label - 1** |
| Logistic Regression | C=0.5, class_weight= 'balanced' | 0.855 | 0.971 | 0.248 | 0.811 | 0.720 | 0.883 | 0.369 |
| Random Forest Classifier | default | 0.770 | 0.920 | 0.000 | 1.000 | 0.000 | 0.958 | 0.000 |
|  | n_estimators= 300 | 0.811 | 0.920 | 0.000 | 1.000 | 0.000 | 0.958 | 0.000 |
|  | n_estimators= 500 | 0.812 | 0.920 | 0.000 | 1.000 | 0.000 | 0.958 | 0.000 |
|  | max_depth=3 | 0.732 | 0.920 | 0.000 | 1.000 | 0.000 | 0.958 | 0.000 |
|  | max_depth=3, n_estimators= 300 | 0.741 | 0.920 | 0.000 | 1.000 | 0.000 | 0.958 | 0.000 |
|  | max_depth=3, n_estimators= 500 | 0.741 | 0.920 | 0.000 | 1.000 | 0.000 | 0.958 | 0.000 |
| XGB Classifier | default | 0.808 | 0.952 | 0.256 | 0.875 | 0.493 | 0.912 | 0.337 |
|  | n_estimators=100 | 0.808 | 0.952 | 0.256 | 0.875 | 0.493 | 0.912 | 0.337 |
|  | n_estimators=300 | 0.804 | 0.939 | 0.313 | 0.943 | 0.300 | 0.941 | 0.306 |
|  | n_estimators=500 | 0.807 | 0.934 | 0.351 | 0.964 | 0.221 | 0.949 | 0.271 |
|  | max_depth=3 | 0.829 | 0.966 | 0.219 | 0.788 | 0.685 | 0.868 | 0.332 |
|  | max_depth=3, n_estimators= 100 | 0.829 | 0.966 | 0.219 | 0.788 | 0.685 | 0.868 | 0.332 |
|  | max_depth=3, n_estimators= 300 | 0.821 | 0.959 | 0.255 | 0.853 | 0.579 | 0.903 | 0.354 |
|  | max_depth=3, n_estimators= 500 | 0.814 | 0.951 | 0.271 | 0.889 | 0.479 | 0.919 | 0.346 |
| MLP Classifier | early_stopping=True, max_iter= 300 | 0.847 | 0.931 | 0.601 | 0.991 | 0.158 | 0.960 | 0.250 |
|  | early_stopping=True, hidden_layer_sizes= (100,50,25), max_iter= 300 | 0.849 | 0.925 | 0.657 | 0.997 | 0.073 | 0.960 | 0.132 |
| SVC | cache_size= 5000, class_weight= 'balanced', kernel= 'linear', probability= True | 0.828 | 0.956 | 0.287 | 0.886 | 0.526 | 0.920 | 0.371 |
|  | C=0.5, cache_size= 5000, class_weight= 'balanced', kernel= 'linear', probability= True | 0.840 | 0.964 | 0.259 | 0.842 | 0.637 | 0.899 | 0.368 |
|  | cache_size=5000, class_weight= 'balanced', kernel=’rbf’, probability= True | 0.849 | 0.939 | 0.484 | 0.975 | 0.270 | 0.957 | 0.346 |
|  | C=0.5, cache_size= 5000, class_weight= 'balanced', kernel=’rbf’, probability= True | 0.853 | 0.953 | 0.377 | 0.933 | 0.468 | 0.943 | 0.418 |

**Table S14.** The performance of machine learning models under varying hyperparameters on the MIMIC dataset (**original dataset**). The default hyperparameters from the scikit-learn library are used unless specifically stated under 'Parameters.' The comparatively best ROC-AUC value for each machine learning model is highlighted in blue. For brevity, the results corresponding to only **BioClinical ModernBERT** feature representation are mentioned here.

| **Classifier** | **Parameters** | **AUC ROC** | **Precision** | | **Recall** | | **F1-Score** | |
| --- | --- | --- | --- | --- | --- | --- | --- | --- |
|  |  |  | **Class 0** | **Class 1** | **Class 0** | **Class 1** | **Class 0** | **Class 1** |
| SVC | cache_size=5000, class_weight='balanced', kernel= 'linear', probability= True | 0.814 | 0.973 | 0.194 | 0.725 | 0.765 | 0.830 | 0.310 |
|  | C=0.5, cache_size= 5000, class_weight= 'balanced', kernel= 'linear', probability= True | 0.814 | 0.973 | 0.194 | 0.725 | 0.764 | 0.831 | 0.310 |
|  | cache_size= 5000, class_weight= 'balanced', kernel=’rbf’, probability= True | 0.757 | 0.975 | 0.133 | 0.523 | 0.845 | 0.681 | 0.231 |
|  | C=0.5, cache_size= 5000, class_weight= 'balanced', kernel=’rbf’, probability= True | 0.726 | 0.973 | 0.118 | 0.444 | 0.860 | 0.609 | 0.208 |
| Logistic Regression | C=0.5, class_weight= 'balanced' | 0.811 | 0.973 | 0.186 | 0.706 | 0.773 | 0.818 | 0.300 |
| Random Forest Classifier | default | 0.762 | 0.920 | 1.000 | 1.000 | 0.001 | 0.958 | 0.002 |
|  | n_estimators= 300 | 0.766 | 0.920 | 1.000 | 1.000 | 0.001 | 0.958 | 0.002 |
|  | n_estimators= 500 | 0.770 | 0.920 | 0.000 | 1.000 | 0.000 | 0.958 | 0.000 |
|  | max_depth= 3 | 0.750 | 0.920 | 0.000 | 1.000 | 0.000 | 0.958 | 0.000 |
|  | max_depth= 3, n_estimators= 300 | 0.754 | 0.920 | 0.000 | 1.000 | 0.000 | 0.958 | 0.000 |
|  | max_depth= 3, n_estimators= 500 | 0.750 | 0.920 | 0.000 | 1.000 | 0.000 | 0.958 | 0.000 |
| XGB Classifier | default | 0.795 | 0.942 | 0.300 | 0.930 | 0.345 | 0.936 | 0.321 |
|  | n_estimators= 100 | 0.795 | 0.942 | 0.300 | 0.930 | 0.345 | 0.936 | 0.321 |
|  | n_estimators= 300 | 0.806 | 0.932 | 0.427 | 0.979 | 0.178 | 0.955 | 0.252 |
|  | n_estimators= 500 | 0.812 | 0.931 | 0.457 | 0.984 | 0.157 | 0.957 | 0.234 |
|  | max_depth= 3 | 0.815 | 0.965 | 0.205 | 0.770 | 0.682 | 0.857 | 0.315 |
|  | max_depth= 3, n_estimators= 100 | 0.815 | 0.965 | 0.205 | 0.770 | 0.682 | 0.857 | 0.315 |
|  | max_depth= 3, n_estimators= 300 | 0.807 | 0.957 | 0.245 | 0.851 | 0.558 | 0.901 | 0.340 |
|  | max_depth= 3, n_estimators= 500 | 0.807 | 0.951 | 0.274 | 0.891 | 0.466 | 0.921 | 0.345 |
| MLPClassifier | early_stopping= True, max_iter= 300 | 0.805 | 0.921 | 0.750 | 1.000 | 0.007 | 0.959 | 0.013 |
|  | early_stopping= True, hidden_layer_sizes= (100,50,25), max_iter= 300 | 0.809 | 0.922 | 0.551 | 0.998 | 0.030 | 0.959 | 0.058 |

**Table S15.** The performance of machine learning models under varying hyperparameters on the MIMIC dataset with **5% error-rate**. The default hyperparameters from the scikit-learn library are used unless specifically stated under 'Parameters.' The comparatively best ROC-AUC value for each machine learning model is highlighted in blue. For brevity, the results corresponding to only **BioClinical ModernBERT** feature representation are mentioned here.

| **Classifier** | **Parameters** | **AUC ROC** | **Precision** | | **Recall** | | **F1 Score** | |
| --- | --- | --- | --- | --- | --- | --- | --- | --- |
|  |  |  | **Class 0** | **Class 1** | **Class 0** | **Class 1** | **Class 0** | **Class 1** |
| SVC | cache_size= 5000, class_weight= 'balanced', kernel= 'linear', probability= True | 0.814 | 0.972 | 0.195 | 0.726 | 0.763 | 0.831 | 0.310 |
|  | C=0.5, cache_size= 5000, class_weight= 'balanced', kernel= 'linear', probability= True | 0.814 | 0.972 | 0.194 | 0.725 | 0.763 | 0.831 | 0.310 |
|  | cache_size= 5000, class_weight= 'balanced', probability= True | 0.765 | 0.974 | 0.137 | 0.541 | 0.836 | 0.696 | 0.235 |
|  | C=0.5, cache_size= 5000, class_weight= 'balanced', probability= True | 0.737 | 0.973 | 0.124 | 0.478 | 0.847 | 0.641 | 0.216 |
| Logistic Regression | C=0.5, class_weight= 'balanced' | 0.810 | 0.972 | 0.183 | 0.700 | 0.771 | 0.814 | 0.295 |
| Random Forest Classifier | default | 0.753 | 0.920 | 1.000 | 1.000 | 0.001 | 0.958 | 0.002 |
|  | n_estimators= 300 | 0.763 | 0.920 | 0.000 | 1.000 | 0.000 | 0.958 | 0.000 |
|  | n_estimators= 500 | 0.769 | 0.920 | 0.000 | 1.000 | 0.000 | 0.958 | 0.000 |
|  | max_depth= 3 | 0.750 | 0.920 | 0.000 | 1.000 | 0.000 | 0.958 | 0.000 |
|  | max_depth= 3, n_estimators= 300 | 0.750 | 0.920 | 0.000 | 1.000 | 0.000 | 0.958 | 0.000 |
|  | max_depth= 3, n_estimators= 500 | 0.748 | 0.920 | 0.000 | 1.000 | 0.000 | 0.958 | 0.000 |
| XGB Classifier | Default | 0.797 | 0.940 | 0.286 | 0.932 | 0.314 | 0.936 | 0.299 |
|  | n_estimators= 100 | 0.797 | 0.940 | 0.286 | 0.932 | 0.314 | 0.936 | 0.299 |
|  | n_estimators= 300 | 0.807 | 0.930 | 0.374 | 0.977 | 0.157 | 0.953 | 0.221 |
|  | n_estimators= 500 | 0.813 | 0.929 | 0.380 | 0.981 | 0.132 | 0.955 | 0.196 |
|  | max_depth= 3 | 0.811 | 0.965 | 0.200 | 0.764 | 0.678 | 0.853 | 0.309 |
|  | max_depth= 3, n_estimators= 100 | 0.811 | 0.965 | 0.200 | 0.764 | 0.678 | 0.853 | 0.309 |
|  | max_depth= 3, n_estimators= 300 | 0.809 | 0.957 | 0.246 | 0.851 | 0.561 | 0.901 | 0.342 |
|  | max_depth= 3, n_estimators= 500 | 0.808 | 0.950 | 0.263 | 0.889 | 0.456 | 0.918 | 0.333 |
| MLP Classifier | early_stopping= True, max_iter= 300 | 0.818 | 0.927 | 0.525 | 0.992 | 0.107 | 0.958 | 0.178 |
|  | early_stopping= True, hidden_layer_sizes= (100,50,25), max_iter= 300 | 0.823 | 0.926 | 0.658 | 0.996 | 0.085 | 0.960 | 0.150 |

**Table S16.** The performance of machine learning models under varying hyperparameters on the MIMIC dataset with **10% error-rate**. The default hyperparameters from the scikit-learn library are used unless specifically stated under 'Parameters.' The comparatively best ROC-AUC value for each machine learning model is highlighted in blue. For brevity, the results corresponding to only **BioClinical ModernBERT** feature representation are mentioned here.

| **Classifier** | **Parameters** | **AUC ROC** | **Precision** | | **Recall** | | **F1-Score** | |
| --- | --- | --- | --- | --- | --- | --- | --- | --- |
|  |  |  | **Class 0** | **Class 1** | **Class 0** | **Class 1** | **Class 0** | **Class 1** |
| SVC | cache_size= 5000, class_weight= 'balanced', kernel=' linear', probability= True | 0.804 | 0.971 | 0.189 | 0.720 | 0.753 | 0.827 | 0.303 |
|  | C=0.5, cache_size= 5000, class_weight= 'balanced', kernel= 'linear', probability= True | 0.804 | 0.971 | 0.190 | 0.720 | 0.754 | 0.827 | 0.303 |
|  | cache_size= 5000, class_weight= 'balanced', kernel=’rbf’, probability= True | 0.762 | 0.974 | 0.137 | 0.543 | 0.832 | 0.697 | 0.235 |
|  | C=0.5, cache_size= 5000, class_weight= 'balanced', kernel=’rbf’, probability= True | 0.734 | 0.974 | 0.124 | 0.477 | 0.851 | 0.640 | 0.216 |
| Logistic Regression | C=0.5, class_weight= 'balanced' | 0.810 | 0.974 | 0.180 | 0.690 | 0.786 | 0.807 | 0.293 |
| Random Forest Classifier | Default | 0.755 | 0.920 | 0.333 | 1.000 | 0.001 | 0.958 | 0.002 |
|  | n_estimators= 300 | 0.767 | 0.920 | 0.333 | 1.000 | 0.001 | 0.958 | 0.002 |
|  | n_estimators= 500 | 0.767 | 0.920 | 0.333 | 1.000 | 0.001 | 0.958 | 0.002 |
|  | max_depth=3 | 0.745 | 0.920 | 0.000 | 1.000 | 0.000 | 0.958 | 0.000 |
|  | max_depth=3, n_estimators= 300 | 0.749 | 0.920 | 0.000 | 1.000 | 0.000 | 0.958 | 0.000 |
|  | max_depth=3, n_estimators= 500 | 0.748 | 0.920 | 0.000 | 1.000 | 0.000 | 0.958 | 0.000 |
| XGB Classifier | Default | 0.791 | 0.942 | 0.306 | 0.934 | 0.337 | 0.938 | 0.321 |
|  | n_estimators= 100 | 0.791 | 0.942 | 0.306 | 0.934 | 0.337 | 0.938 | 0.321 |
|  | n_estimators= 300 | 0.801 | 0.930 | 0.400 | 0.980 | 0.156 | 0.954 | 0.224 |
|  | n_estimators= 500 | 0.809 | 0.929 | 0.419 | 0.984 | 0.136 | 0.956 | 0.206 |
|  | max_depth=3 | 0.805 | 0.963 | 0.196 | 0.763 | 0.666 | 0.851 | 0.303 |
|  | max_depth=3, n_estimators= 100 | 0.805 | 0.963 | 0.196 | 0.763 | 0.666 | 0.851 | 0.303 |
|  | max_depth=3, n_estimators= 300 | 0.801 | 0.954 | 0.232 | 0.847 | 0.532 | 0.898 | 0.323 |
|  | max_depth=3, n_estimators= 500 | 0.795 | 0.948 | 0.257 | 0.889 | 0.441 | 0.918 | 0.325 |
| MLP Classifier | early_stopping= True, max_iter= 300 | 0.760 | 0.921 | 0.333 | 0.998 | 0.012 | 0.958 | 0.024 |
|  | early_stopping= True, hidden_layer_sizes= (100,50,25), max_iter= 300 | 0.817 | 0.926 | 0.619 | 0.996 | 0.079 | 0.959 | 0.140 |

**Table S17.** The performance of machine learning models under varying hyperparameters on the MIMIC dataset with **15% error-rate**. The default hyperparameters from the scikit-learn library are used unless specifically stated under 'Parameters.' The comparatively best ROC-AUC value for each machine learning model is highlighted in blue. For brevity, the results corresponding to only **BioClinical ModernBERT** feature representation are mentioned here.

| **Classifier** | **Parameters** | **AUC ROC** | **Precision** | | **Recall** | | **F1-Score** | |
| --- | --- | --- | --- | --- | --- | --- | --- | --- |
|  |  |  | **Class 0** | **Class 1** | **Class 0** | **Class 1** | **Class 0** | **Class 1** |
| SVC | cache_size= 5000, class_weight= 'balanced', kernel= 'linear', probability= True | 0.808 | 0.972 | 0.189 | 0.716 | 0.760 | 0.825 | 0.302 |
|  | C=0.5, cache_size= 5000, class_weight= 'balanced', kernel= 'linear', probability= True | 0.808 | 0.972 | 0.188 | 0.716 | 0.760 | 0.824 | 0.302 |
|  | cache_size= 5000, class_weight= 'balanced', kernel=’rbf’, probability= True | 0.762 | 0.975 | 0.137 | 0.542 | 0.839 | 0.696 | 0.236 |
|  | C=0.5, cache_size= 5000, class_weight= 'balanced', kernel=’rbf’, probability= True | 0.735 | 0.974 | 0.124 | 0.474 | 0.855 | 0.637 | 0.216 |
| Logistic Regression | C=0.5, class_weight= 'balanced' | 0.806 | 0.972 | 0.184 | 0.703 | 0.770 | 0.816 | 0.297 |
| Random Forest Classifier | Default | 0.756 | 0.920 | 0.400 | 1.000 | 0.002 | 0.958 | 0.004 |
|  | n_estimators= 300 | 0.770 | 0.920 | 0.333 | 1.000 | 0.001 | 0.958 | 0.002 |
|  | n_estimators= 500 | 0.766 | 0.920 | 0.000 | 1.000 | 0.000 | 0.958 | 0.000 |
|  | max_depth=3 | 0.744 | 0.920 | 0.000 | 1.000 | 0.000 | 0.958 | 0.000 |
|  | max_depth=3, n_estimators= 300 | 0.751 | 0.920 | 0.000 | 1.000 | 0.000 | 0.958 | 0.000 |
|  | max_depth=3, n_estimators= 500 | 0.751 | 0.920 | 0.000 | 1.000 | 0.000 | 0.958 | 0.000 |
| XGB Classifier | Default | 0.791 | 0.941 | 0.281 | 0.927 | 0.328 | 0.934 | 0.303 |
|  | n_estimators= 100 | 0.791 | 0.941 | 0.281 | 0.927 | 0.328 | 0.934 | 0.303 |
|  | n_estimators= 300 | 0.802 | 0.930 | 0.374 | 0.977 | 0.158 | 0.953 | 0.222 |
|  | n_estimators= 500 | 0.808 | 0.929 | 0.417 | 0.984 | 0.130 | 0.956 | 0.197 |
|  | max_depth= 3 | 0.806 | 0.965 | 0.200 | 0.763 | 0.682 | 0.852 | 0.309 |
|  | max_depth=3, n_estimators= 100 | 0.806 | 0.965 | 0.200 | 0.763 | 0.682 | 0.852 | 0.309 |
|  | max_depth=3, n_estimators= 300 | 0.808 | 0.955 | 0.235 | 0.847 | 0.543 | 0.898 | 0.328 |
|  | max_depth=3, n_estimators= 500 | 0.808 | 0.949 | 0.264 | 0.891 | 0.449 | 0.919 | 0.332 |
| MLP Classifier | early_stopping= True, max_iter= 300 | 0.803 | 0.922 | 0.477 | 0.998 | 0.024 | 0.958 | 0.045 |
|  | early_stopping= True, hidden_layer_sizes= (100,50,25), max_iter= 300 | 0.820 | 0.927 | 0.506 | 0.992 | 0.097 | 0.958 | 0.163 |

**Table S18.** The performance of machine learning models under varying hyperparameters on the MIMIC dataset with **20% error-rate**. The default hyperparameters from the scikit-learn library are used unless specifically stated under 'Parameters.' The comparatively best ROC-AUC value for each machine learning model is highlighted in blue. For brevity, the results corresponding to only **BioClinical ModernBERT** feature representation are mentioned here.

| **Classifier** | **Parameters** | **AUC ROC** | **Precision** | | **Recall** | | **F1-Score** | |
| --- | --- | --- | --- | --- | --- | --- | --- | --- |
|  |  |  | **Class 0** | **Class 1** | **Class 0** | **Class 1** | **Class 0** | **Class 1** |
| SVC | cache_size= 5000, class_weight= 'balanced', kernel= 'linear', probability= True | 0.808 | 0.973 | 0.192 | 0.721 | 0.766 | 0.828 | 0.308 |
|  | C=0.5, cache_size= 5000, class_weight= 'balanced', kernel= 'linear', probability= True | 0.808 | 0.973 | 0.192 | 0.721 | 0.766 | 0.828 | 0.308 |
|  | cache_size= 5000, class_weight= 'balanced', kernel=’rbf’, probability= True | 0.760 | 0.973 | 0.136 | 0.542 | 0.830 | 0.696 | 0.234 |
|  | C=0.5, cache_size= 5000, class_weight= 'balanced', kernel=’rbf’, probability= True | 0.734 | 0.974 | 0.123 | 0.472 | 0.854 | 0.636 | 0.215 |
| Logistic Regression | C=0.5, class_weight= 'balanced' | 0.806 | 0.973 | 0.181 | 0.696 | 0.774 | 0.811 | 0.293 |
| Random Forest Classifier | Default | 0.756 | 0.920 | 0.750 | 1.000 | 0.003 | 0.958 | 0.007 |
|  | n_estimators= 300 | 0.764 | 0.920 | 0.000 | 1.000 | 0.000 | 0.958 | 0.000 |
|  | n_estimators= 500 | 0.765 | 0.920 | 1.000 | 1.000 | 0.002 | 0.958 | 0.005 |
|  | max_depth=3 | 0.746 | 0.920 | 0.000 | 1.000 | 0.000 | 0.958 | 0.000 |
|  | max_depth=3, n_estimators= 300 | 0.750 | 0.920 | 0.000 | 1.000 | 0.000 | 0.958 | 0.000 |
|  | max_depth=3, n_estimators= 500 | 0.748 | 0.920 | 0.000 | 1.000 | 0.000 | 0.958 | 0.000 |
| XGB Classifier | Default | 0.781 | 0.942 | 0.300 | 0.931 | 0.339 | 0.937 | 0.318 |
|  | n_estimators= 100 | 0.781 | 0.942 | 0.300 | 0.931 | 0.339 | 0.937 | 0.318 |
|  | n_estimators= 300 | 0.792 | 0.930 | 0.390 | 0.979 | 0.157 | 0.954 | 0.224 |
|  | n_estimators= 500 | 0.798 | 0.929 | 0.421 | 0.984 | 0.132 | 0.956 | 0.201 |
|  | max_depth= 3 | 0.791 | 0.962 | 0.193 | 0.765 | 0.648 | 0.852 | 0.297 |
|  | max_depth=3, n_estimators= 100 | 0.791 | 0.962 | 0.193 | 0.765 | 0.648 | 0.852 | 0.297 |
|  | max_depth=3, n_estimators= 300 | 0.787 | 0.954 | 0.230 | 0.844 | 0.536 | 0.896 | 0.322 |
|  | max_depth=3, n_estimators= 500 | 0.788 | 0.948 | 0.259 | 0.891 | 0.440 | 0.919 | 0.326 |
| MLPClassifier | early_stopping= True, max_iter= 300 | 0.794 | 0.921 | 0.560 | 0.999 | 0.016 | 0.958 | 0.031 |
|  | early_stopping= True, hidden_layer_sizes= (100,50,25), max_iter= 300 | 0.817 | 0.929 | 0.487 | 0.988 | 0.130 | 0.958 | 0.205 |
